# Robust Photocatalytic MICROSCAFS^®^ with Interconnected Macropores for Sustainable Solar-Driven Water Purification

**DOI:** 10.3390/ijms25115958

**Published:** 2024-05-29

**Authors:** Mário Vale, Beatriz T. Barrocas, Rita M. N. Serôdio, M. Conceição Oliveira, José M. Lopes, Ana C. Marques

**Affiliations:** 1 Centro de Recursos Naturais e Ambiente (CERENA), Chemical Engineering Department, Instituto Superior Técnico, University of Lisbon, Av. Rovisco Pais, 1049-001 Lisbon, Portugal; mario.vale@tecnico.ulisboa.pt (M.V.); beatriz.trindade.barrocas@tecnico.ulisboa.pt (B.T.B.); rita.serodio@tecnico.ulisboa.pt (R.M.N.S.); 2 Centro de Desenvolvimento de Produto e Transferência de Tecnologia (CDP2T), Escola Superior de Tecnologia de Setúbal, Instituto Politécnico de Setúbal, 2910-761 Setúbal, Portugal; 3 Centro de Física e Engenharia de Materiais Avançados (CeFEMA), Instituto Superior Técnico, University of Lisbon, 1049-001 Lisbon, Portugal; 4 Centro de Química Estrutural (CQE), Chemical Engineering Department, Institute of Molecular Sciences, Instituto Superior Técnico, University of Lisbon, Av. Rovisco Pais, 1049-001 Lisbon, Portugal; conceicao.oliveira@tecnico.ulisboa.pt (M.C.O.); jmlopes@tecnico.ulisboa.pt (J.M.L.)

**Keywords:** microspheres, macroporosity, sol–gel, titania, heterogeneous photocatalysis, kinetics

## Abstract

Advanced oxidation processes, including photocatalysis, have been proven effective at organic dye degradation. Tailored porous materials with regulated pore size, shape, and morphology offer a sustainable solution to the water pollution problem by acting as support materials to grafted photocatalytic nanoparticles (NPs). This research investigated the influence of pore and particle sizes of photocatalytic MICROSCAFS^®^ on the degradation of methyl orange (MO) in aqueous solution (10 mg/L). Photocatalytic MICROSCAFS^®^ are made of binder-less supported P25 TiO_2_ NPs within MICROSCAFS^®^, which are silica–titania microspheres with a controlled size and interconnected macroporosity, synthesized by an adapted sol–gel method that involves a polymerization-induced phase separation process. Photocatalytic experiments were performed both in batch and flow reactors, with this latter one targeting a proof of concept for continuous transformation processes and real-life conditions. Photocatalytic degradation of 87% in 2 h (batch) was achieved, using a calibrated solar light simulator (1 sun) and a photocatalyst/pollutant mass ratio of 23. This study introduces a novel flow kinetic model which provides the modeling and simulation of the photocatalytic MICROSCAFS^®^ performance. A scavenger study was performed, enabling an in-depth mechanistic understanding. Finally, the transformation products resulting from the MO photocatalytic degradation were elucidated by high-resolution mass spectrometry experiments and subjected to an in silico toxicity assessment.

## 1. Introduction

Water pollution is one of the biggest ecological problems that humanity currently faces. Synthetic dyes are used in many industries, such as textile processing, the pharmaceutical sector, and food production, and approximately 80% of the resulting wastewaters are released untreated into the environment [1].

Water remediation is therefore one of the priorities in present-day scientific research. Among the explored methods for this purpose, heterogeneous photocatalysis offers many advantages, such as a low cost, high flexibility of the process, the use of natural energy sources, relatively low quantity of required catalyst, and, depending on the photocatalyst’s nature, high physical stability and recyclability of the materials employed, which make it an environmentally friendly technique [2,3,4]. However, the typical nanometric size of the photocatalysts, when used unsupported, in the powder form, makes their removal complex after the reaction, which is a significant drawback of this technique, being currently among the major limitations for the application of photocatalysis in a real-life scenario.

One solution for facilitating the photocatalysts recovery is their immobilization, preferably by grafting, into inert supports of relatively greater proportions. Binder-less processes enable us to overpass issues regarding the decrease in reactive sites due to hydrophobic effects and low surface area that result from organic or inorganic binders [5]. They are also preferred to achieve stable supported photocatalysts. 

Undoped TiO_2_ NPs such as Aeroxide P25 are still a viable photocatalyst according to today’s standards, due to its low cost and high commercial availability. TiO_2_ NPs have been supported in multiple materials, like biochar [6], silica spheres [7], natural clays [8], cellulose nanofibrils [9], poly(methyl methacrylate) (PMMA) [10] and polyvinyl alcohol (PVA) films [11], and fly ash [12], to degrade MO. Additionally, TiO_2_ aerogel was deposited over silica-coated bacterial nanocellulose scaffolds with relatively fast photocatalytic degradation of methylene blue, using UV-Vis light in a continuous flow reactor [13].

The majority of reported photocatalytic experiments is still made in batch conditions, which have some drawbacks if a real-life application is envisioned, like finite volume and non-continuous processing. Seldomly reported, the photocatalysts are susceptible to mechanical stress and eventual fracture caused by the stirring in the batch reactor, generating smaller particles and a higher surface area, which overestimates the photocatalytic performance and jeopardizes the claimed easy removal of the photocatalysts after a test. Also, porosity data, like specific surface area and pore diameter, are not properly discussed or even totally or partially missing in some papers in the literature [14,15,16,17,18,19,20,21,22], neglecting possible synergies and effects granted by the catalytic supports in what regards, e.g., mass transfer. Concerning the particle size, most of the reported microspherical supports are usually very small, ranging from 0.5 to 3 µm [18,19,20,21,23,24,25], which may impose problems and extra complexity and costs in a real-life application, in what regards the setup and recovery aspects.

Lucchini et al. [9] employed a continuous-flow reactor design, with total recirculation to a tank, consisting of a cylindrical PMMA cell topped by a quartz window, where the simulated sunlight impinges at a constant irradiance. The reactor chamber was filled with a cellulose nanofibril (CNF)-based monolith loaded with synthesized TiO_2_ NPs for the degradation of MO and paracetamol in liquid solution, which were made to circulate in a tube system connected to the bottom of the chamber and the tank. Then, in our previous study reported by Marques et al. [7], we applied the same reactor design and procedure using photocatalytic silica microspheres instead of a CNF monolith. These silica microspheres, namely SiO_2_ MICROSCAFS^®^, were for the first time applied as supports for synthesized Trizma^®^-functionalized TiO_2_ NPs. Their inorganic nature provides them with high thermal and chemical stability. 

Herein, we study in detail the effect of pore and particle sizes of supported photocatalytic systems, called photocatalytic MICROSCAFS^®^, in batch and continuous flow-type reactors, on the photocatalytic degradation of MO in aqueous solution (10 mg/L). The continuous flow reactor was used solely as proof of concept for the use of the new photocatalysts in continuous transformation processes. The MICROSCAFS^®^ in the present work are made of a multicomponent oxide composition, silica–titania (SiO_2_-TiO_2_ at ca. 80–20% (molar)), and were obtained via a sol–gel reaction inside the water droplets of a water-in-oil emulsion (W/O). As before, the tailored interconnected macroporosity of the MICROSCAFS^®^ was achieved through polymerization-induced phase separation [26,27,28]. The resulting MICROSCAFS^®^ were loaded with well-known commercial P25 TiO_2_ NPs, using a simple binder-less process in which the NPs were at first physically entrapped inside the porosity of the MICROSCAFS^®^, forming a layer on the surface and inner pores of the full particle, followed by a covalent binding promoted by heat treatment. Therefore, the photocatalytic MICROSCAFS^®^ used in this study are composed of MICROSCAFS^®^ with immobilized photocatalytic P25 NPs. These latter ones are the active phase of our photocatalytic system. A similar preparation was followed in our previous paper, including additional gold nanoparticles, but, contrary to the present paper, only addressed a batch reactor for photocatalysis experiments [29]. Moreover, a new kinetic model for the flow reactor is herein proposed, and the solar light-driven degradation of MO using the photocatalytic MICROSCAFS^®^ is simulated using the proposed model and compared with experimental results. Finally, the MO degradation is followed by liquid chromatography–tandem high-resolution mass spectrometry (LC-HRMS/MS) to identify and characterize its by-products, thus providing a deeper understanding of the degradation process.

## 2. Results and Discussion

### 2.1. Characterization of the MICROSCAFS^®^ before and after Heat Treatment and Photocatalyst (P25 TiO_2_ NPs) Loading

The MICROSCAFS^®^ of silica–titania composition are herein shown to be a versatile type of materials that can be obtained with high reproducibility via a relatively simple two-step, fast, low-energy, adapted sol–gel process. Their final characteristics, like pore and particle diameter, and mechanical properties have been recently reported to be easily tuned by changing the synthesis parameters [27]. In this work, we aim to assess the effect of pores size and particles diameter on the photocatalytic performance of P25 TiO_2_ NPs-loaded MICROSCAFS^®^.

**Figure 1:**
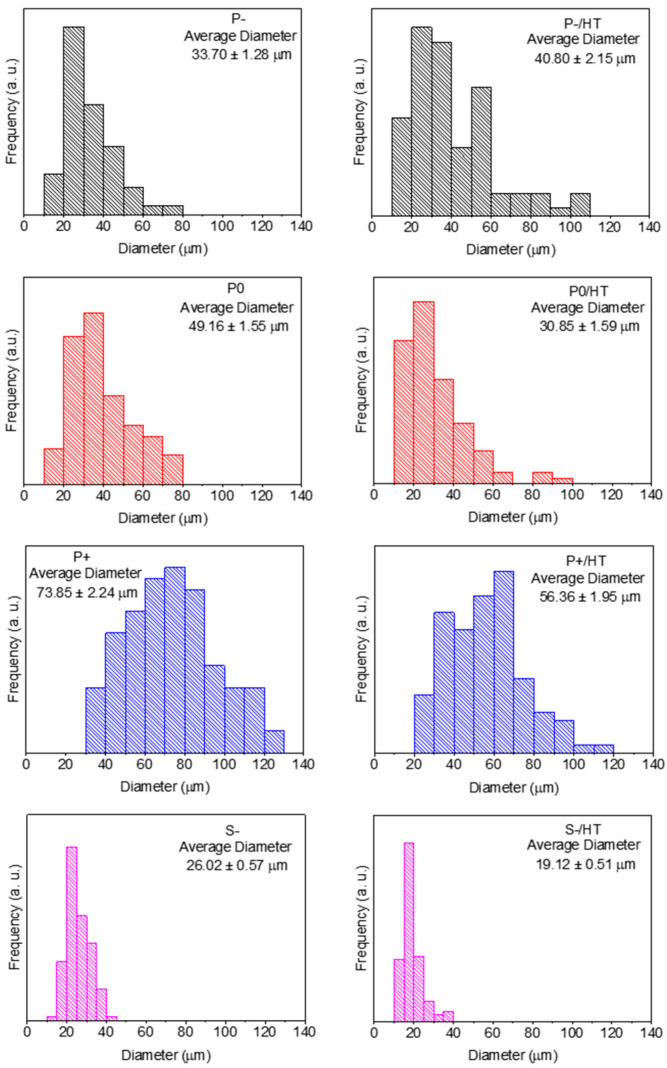
Particle size distributions of the MICROSCAFS^®^ before (**left**) and after HT (**right**) at 900 °C during 30 min.

All the MICROSCAFS^®^’ particle size distributions before and after calcination (HT) are shown in Figure 1, obtained from the SEM images present in Supplementary Figure S3. The average particle diameter tends to increase with the quantity of GPTMS, and the span or the dispersion was found to decrease, particularly when using +25% GPTMS (Supplementary Table S1). GPTMS precursor slows the condensation reaction of the titania and silica precursors due to steric hindrance caused by the relatively big aliphatic chain. This might provide a steady formation of siloxanes and Si-O-Ti bonds inside the water droplets of the emulsion, producing particles with lower size dispersions. The diameters of S− particles were mostly within the range from 20 to 40 µm, which was expected due to the sieving procedure applied in this sample. As expected, after the first HT, most of the samples decreased in size, with sample P− being the only exception. Despite a non-linear change in the average diameter, the particle diameter mode did not change, meaning that the HT had no significant effect on its size, and the difference in the distribution might be due to sampling heterogeneity. This fact is linked to the lower porosity of the P− samples, demonstrated below, so that there are less pores to collapse during calcination. 

Optical microscopy images of the P25 TiO_2_ NPs-loaded and -unloaded MICROSCAFS^®^ used in this work are presented in Supplementary Figure S4. A concentration of P25 TiO_2_ NPs equal to ~23 wt% was achieved in most samples, except for sample S−/HT/P25, which achieved 22 wt%, and sample P−/HT/P25, where such concentration corresponds to 25 wt%, being very close to the nominal concentration at 26 wt%. The different colors of the MICROSCAFS^®^ after the HT are a direct result of the internal porosity of the samples, dictated by the different content of TEOS and GPTMS (Supplementary Figure S4). Spheres with smaller pore sizes, namely P− sample, exhibit a darker color because of the entrapment inside the pore network of generated gases resulting from organics degradation.

**Figure 2:**
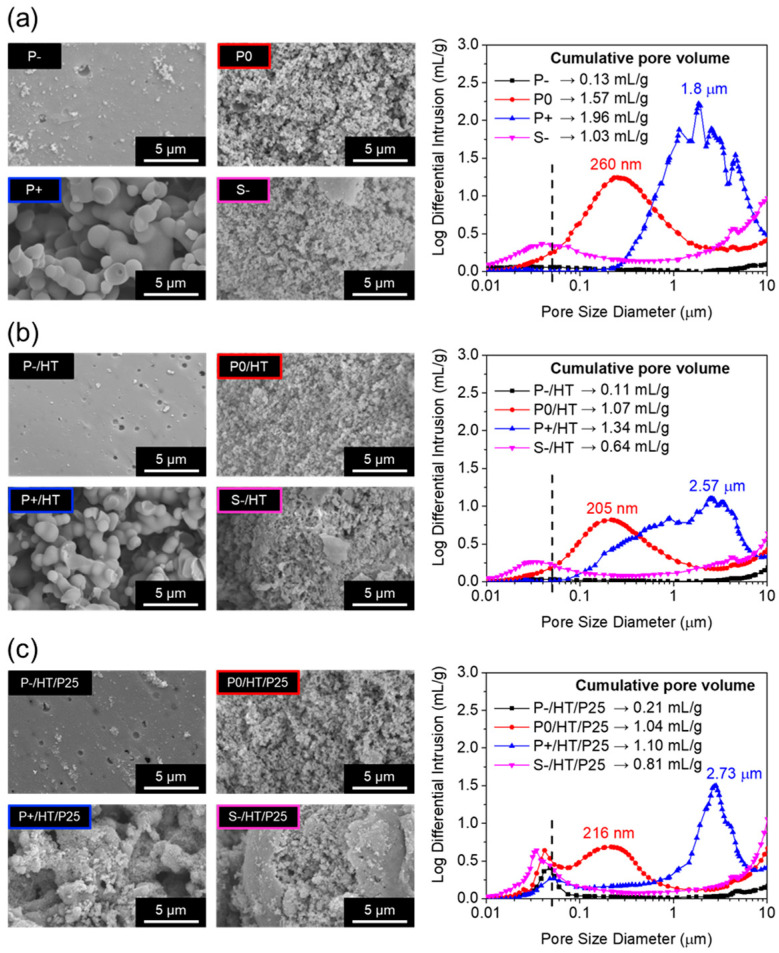
SEM images of the internal porosity (**left**) and corresponding MIP pore size distributions (**right**) of the MICROSCAFS^®^ (**a**) dried at 45 °C, (**b**) heat-treated at 900 °C, and (**c**) loaded with P25 TiO_2_ NPs and heat-treated at 500 °C.

The porosity exhibited by the dried, heat-treated, and P25 TiO_2_ NPs-loaded MICROSCAFS^®^ was analyzed using SEM, MIP (Figure 2), and N_2_ adsorption–desorption isotherms (Supplementary Figure S5). A higher quantity of GPTMS precursor yielded spherical particles with significantly greater macropore size and cumulative pore volume (Figure 2a), which means that the domains of the separated phases, oxide-based xerogel-rich phase (containing Si-O-Si, Si-O-Ti and Ti-O-Ti bonds) and water-rich phase, are larger. The presence of GPTMS simultaneously affected the phase separation and condensation processes, without requiring any phase-separation inducer [27]. The N_2_ adsorption isotherms of all the dried MICROSCAFS^®^ were of type III (IUPAC classification), further corroborating, in the present case, their macroporous morphology, exhibiting specific surface areas (SSAs) that ranged from 4.19 to 46.10 m^2^/g (Supplementary Figure S5a). Samples S− and P0 exhibit the largest SSAs, suggesting the presence of mesopores, which is in agreement with the findings from MIP (Figure 2a). They were both prepared with the same amount of GPTMS; however, sample S− shows less macropores, probably because of the larger content of surfactants (10 g), which might contribute to the disruption of the phase separation due to lower interfacial tension, breaking the skeleton particles as the macropore domains were forming. The presence of Pluronic^®^ P123 is also responsible for the formation of mesopores. Additionally, this sample is found to consist of smaller particles with a rough and fragmented morphology (Supplementary Figures S3 and S4). As expected, after the HT at 900 °C for 30 min, the pore diameters and cumulative pore volumes, as well as the SSA, decreased in almost all the samples (Figure 2b), suggesting the occurrence of some sintering or pore collapse; however, the presence of meso- and macropores is retained (Supplementary Figure S5b). On the other hand, the loading with P25 TiO_2_ NPs resulted in the appearance of a peak at the mesoporosity range, between 30 and 50 nm (Figure 2c), and an increment of the SSA (Supplementary Figure S5c), and did not significantly affect macroporosity. The employed P25 TiO_2_ NPs have an average diameter of about 27.4 ± 0.9 nm (Supplementary Figure S6), so that the interparticle spaces contribute to the mesoporosity exhibited by the P25 TiO_2_ NPs-loaded MICROSCAFS^®^.

As for elemental composition, EDS data in Supplementary Table S2 revealed that the P+ sample is the one with a Si and Ti at. % closer to the nominal Si and Ti precursor molar ratio of 80/20. Also, it is clear that MICROSCAFS^®^ present higher Ti/Si at. % ratios after loading with the P25 TiO_2_ NPs, as expected. Interestingly, the P+ sample, the one exhibiting large interconnected macroporosity, has less loading of P25 TiO_2_ NPs, given by the lower Ti/Si at. % ratio, compared to P− and P0 samples, for the same loading conditions. This might be due to the facilitated flow of the P25 TiO_2_ NPs’ dispersion throughout the large pores and, therefore, less clogging of the pores, which may be beneficial for the wastewater flow throughout the MICROSCAFS^®^ in the photocatalytic tests.

The FTIR spectra of the MICROSCAFS^®^ after drying at 45 °C (Supplementary Figure S7a,b) contain multiple bands characteristic of the oxides’ network, as well as of other organic compounds from the reactional mixture, including some residues of precursors (alkoxides), surfactant and decalin. The band at 1267 cm^−1^ is characteristic of the epoxy group of the GPTMS [30,31]; however, the peaks at 906 and 850 cm^−1^, ascribed to C–O and C–O–C stretching of the GPTMS’ epoxy (oxirane), are hard to detect because they might be hidden by the intense band at 915 cm^−1^ (Si-OH, or Si-O^−^ from Si-O-Ti units). After the heat treatment at 900 °C (Supplementary Figure S7c,d), the organic groups completely disappeared. The intensity of the band at around 934 cm^−1,^ which includes the silanol (OH groups) vibrations, becomes lower, probably because of the OH elimination during heat treatment and some degree of phase separation, i.e., Si-O-Ti conversion to Si-O-Si and Ti-O-Ti links. An intense band at 1065 cm^−1^ is related to Si-O-Si asymmetric stretching vibration. After loading with P25 TiO_2_ NPs (Supplementary Figure S7e,f), the I(Ti-O-Ti, 450 cm^−1^)/I(Si-O-Si, 1065 cm^−1^) ratio increased in all samples (Supplementary Table S3), as expected, meaning that a higher amount of titania is present in the photocatalytic MICROSCAFS^®^. Again, the higher the pore size (P+/HT/P25), the lower the amount of Ti (TiO_2_), thus corroborating the EDS results (Supplementary Table S2).

### 2.2. Photocatalytic Studies on the P25 TiO_2_ NPs-Loaded MICROSCAFS^®^

**Figure 3:**
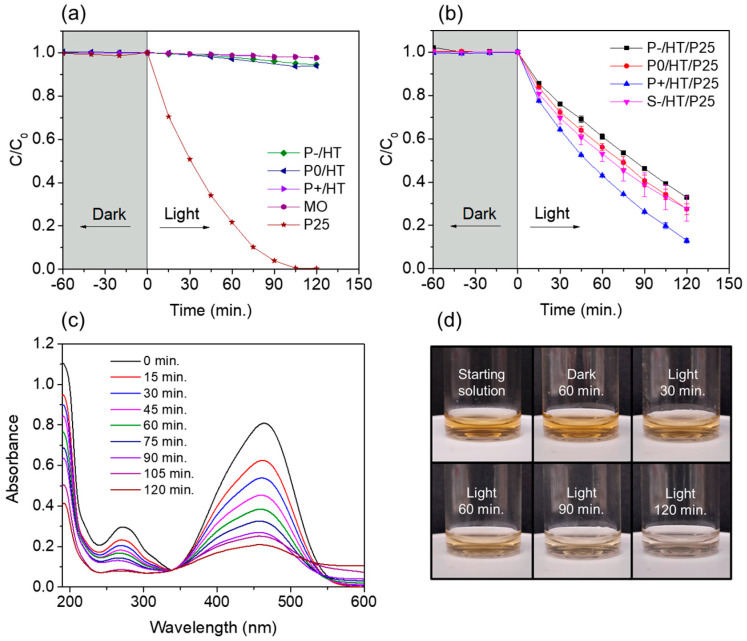
(**a**) Plots of C/C_0_ versus time of the heat-treated unloaded MICROSCAFS^®^, i.e., without photocatalyst (P−/HT, P0/HT, and P+/HT), P25 TiO_2_ NPs (13 mg), and photolysis of the MO dye. (**b**) Plots of C/C_0_ versus time of the photocatalytic P25 TiO_2_ NP-loaded MICROSCAFS^®^. (**c**) UV-vis spectra of MO solution aliquots taken during the light phase of a P+/HT/P25 experiment. (**d**) Photos of MO solution aliquots at the starting of the illumination and after 120 min when using the P+/HT/P25. Experiments performed in batch conditions at 19 °C, pH = 7, mass(TiO_2_ NPs)/mass(MO) = 23, 50 mL of 10 mg/L MO aq. solution, 50 mg of P25 TiO_2_ NPs-loaded MICROSCAFS^®^ (11–12.5 mg TiO_2_), irradiance = 1000 W m^−2^ (1 sun).

The photolysis of MO resulted in no degradation over the 2 h of light exposure, whereas the photocatalytic P25 TiO_2_ NPs (non-supported) were able to fully degrade the pollutant in the same period (Figure 3a). No adsorption was detected for the photocatalytic NPs or for the heat-treated unloaded MICROSCAFS^®^. Also, these latter ones exhibited no significant photocatalytic activity (Figure 3a), which means that TiO_2_ NPs are the active phase of our photocatalytic system. 

Regarding the loaded MICROSCAFS^®^, photocatalytic degradation data using the batch reactor showed that, overall, larger pores proved to be better, with the P+/HT/P25 sample degrading 87% of the MO dye in 2 h of exposure to simulated solar light (Figure 3b). Larger interconnected pores improved the accessibility and the diffusion of both the reactants and products to and from the active sites, speeding up the reaction and promoting the photocatalytic degradation of MO species. Also, similar results are achieved for P0/HT/P25 and S−/HT/P25, which reveals that the difference in size of these particles does not play an important role in the photocatalytic performance. 

The MO UV-Vis spectra had two major absorption bands, around 464 and 271 nm, which are from the aromatic ring in the MO molecule (Figure 3c) [32]. UV-Vis absorption spectra intensity at ~464 nm decreased as the reaction proceeded during light exposure, suggesting some degree of degradation. The characteristic orange color of the solution clearly faded over time with the exposure to the simulated solar light, proving the photocatalytic MICROSCAFS^®^’s efficacy (Figure 3d).

The presence of silica in the MICROSCAFS^®^ hindered the absorption of the photocatalyst (Supplementary Figure S8). Its effect was higher for the P+/HT/P25 sample, which had the highest amount of Si atoms by EDS (Supplementary Table S2). Silicon dioxide is known for its wide bandgap of ca. 9 eV, which plays the role of an energetic barrier for the solar radiation (in particular visible light), thus not generating excited electrons and holes and inhibiting photocatalytic activity [33]. On the other hand, the photocatalytic activity exhibited by the P+/HT/P25 sample was the highest, demonstrating that other factors, such as the presence of wider and interconnected pores, played an important role in the achievement of photocatalytic activity. The presence of pores has been reported to increase the absorbance of the light in, e.g., aerogels [34]. In this case, it has been reported that the pore network traps the photons, which then diffuse over a few millimeters and thus increase the probability of the light to be absorbed. We propose that a similar effect might be happening with our MICROSCAFS^®^. Compared to a dense bead, the macroporous network extends the pathway of the photons, increasing their probability to be absorbed by P25 TiO_2_ NPs located in the inner surface of the pores, thus yielding higher photocatalytic activities.

**Figure 4:**
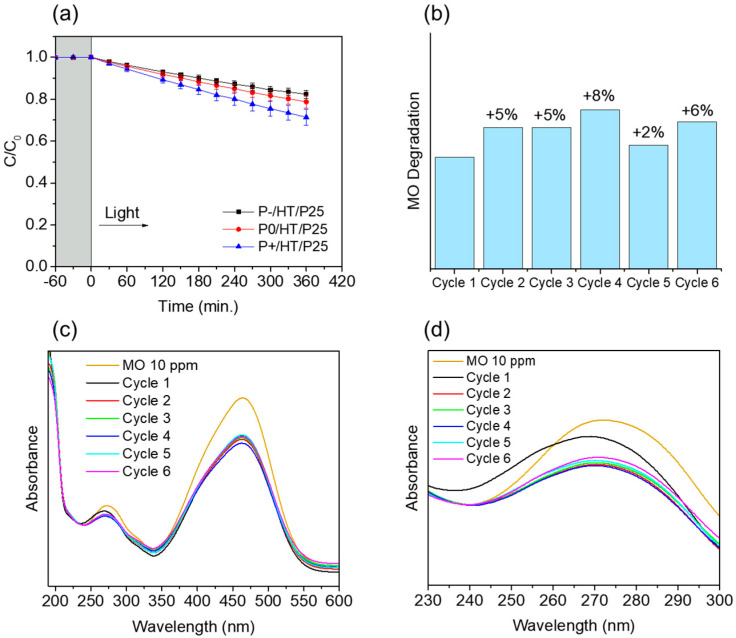
(**a**) Plots of C/C_0_ versus time of the P25 TiO_2_ NPs-loaded MICROSCAFS^®^ using a continuous flow setup. (**b**) MO degradation evolution achieved for 6 consecutive cycles with solar light irradiation for 6 h, using sample P+/HT/P25. (**c**) UV-Vis spectra of the final MO solution after each cycle in a range from 190 to 600 nm. (**d**) UV-Vis spectra of the final MO solution after each cycle in a range from 230 to 300 nm. Experiments performed in flow conditions at 19 °C, pH = 7, mass (P25 TiO_2_ NPs)/mass(MO) = 23, 200 mL of 10 mg/L MO aq. solution, 200 mg of P25 TiO_2_ NPs-loaded MICROSCAFS^®^ (46–50 mg TiO_2_), volumetric flow = 10 mL min^−1^, irradiance = 1000 W m^−2^ (1 sun).

As a proof of concept, the same MICROSCAFS^®^ were employed in a continuous flow reactor with total recirculation to a tank using the same parameters: pH, irradiance, temperature, and photocatalyst/pollutant mass ratio (Figure 4). The volume of the reactor is smaller in this case than in the batch reactor, and it is connected to a tank where no reaction occurs. However, the same tendency was observed as in the batch reactor: the greater the interconnected pores size (P+/HT/P25), the higher the pollutant (MO) degradation. MO suffers a 29% degradation within 6 h (Figure 4a), using P+/HT/P25 as photocatalytic MICROSCAFS^®^.

It should be noted that the reaction was significantly slower than that in the batch reactor, which is explained by the different reactor design. First, the flow reactor allows for a much smaller exposure area (3.14 cm^2^) to solar light than the batch reactor (12.57 cm^2^). In the flow reactor, the pollutant solution passes under the quartz glass, inside the chamber, going through the thin layer of porous photocatalytic MICROSCAFS^®^ (Supplementary Figure S9). Despite the thin layer of MICROSCAFS^®^, i.e., only ca. 1.5 mm thickness, the particles at the top, closer to the quartz glass, are more exposed to the solar light than the particles beneath, whereas in the batch reactor, all the particles are constantly exposed to the light in a free-flow fashion. In contrast, in batch, all the MO solution is in permanent contact with the light and photocatalyst over the full extent of the reaction. Moreover, the fraction of the MO solution exposed to the solar light in the flow setup and, therefore, partially degraded returns to the intercalated tank that contains the MO solution in a higher concentration. On the other hand, the continuous flow system is somewhat similar to a real-case scenario, and it greatly facilitates and enables the recyclability of the photocatalytic MICROSCAFS^®^, preserving their mechanical integrity during numerous cycles (does not involve stirring) and their easy removal/collection. Supplementary Figure S10 shows samples P−/HT/P25 and P+/HT/P25 after a photocatalytic test in flow and batch setups. Compared to the SEM images of Supplementary Figure S3, we can see that the denser (stiffer [27]) sample, P−/HT/P25, is fully preserved whatever the setup employed for photocatalysis, whereas the largely porous P+/HT/P25 MICROSCAFS^®^ are fully preserved after the continuous flow tests but tend to fracture when stirring at a high rate is applied. The higher compressibility, or flexibility, of the P+/HT/P25 spheres did not influence the photocatalytic efficiency because the same trend occurred both in batch and in flow. Sample S−/HT/P25 was not studied in the flow reactor due to its relatively small particle diameter, which clogged the filter employed in the experiment.

As observed in Figure 4b, the final degradation of MO with P+/HT/P25 increased slightly after the first cycle and stayed relatively stable during the subsequent five tested flow cycles. The UV-Vis absorbance spectra in Figure 4c,d were very similar from the second cycle onward, whereas in the first cycle, not only the 271 nm band absorbance is higher, but it is shifted to lower wavelengths, suggesting a (minor) release of TiO_2_ NPs, probably those in excess and not covalently bonded to the MICROSCAFS^®^ surface. Also, the lower MO degradation achieved in the first cycle might have been due to some congestion of the pores of the P+/HT/P25 sample (Figure 4b), which, after the first cycle, does not happen anymore due to the release of the NPs in excess. In this sense, a better flow throughout the MICROSCAFS^®^ and more access to active sites is promoted, leading to the observed better performance after the first cycle. On the other hand, we cannot disregard variability issues, and such a difference in MO degradation for the various cycles could be simply within the error of the analyses. Finally, the flow reactor was more suitable for the recyclability studies than the batch reactor since the same particles stayed inside the reactor in every cycle without the need for any separation process, like centrifugation or filtration, which avoided the loss of some photocatalyst mass inherent to these separation processes.

### 2.3. Kinetics Studies

**Figure 5:**
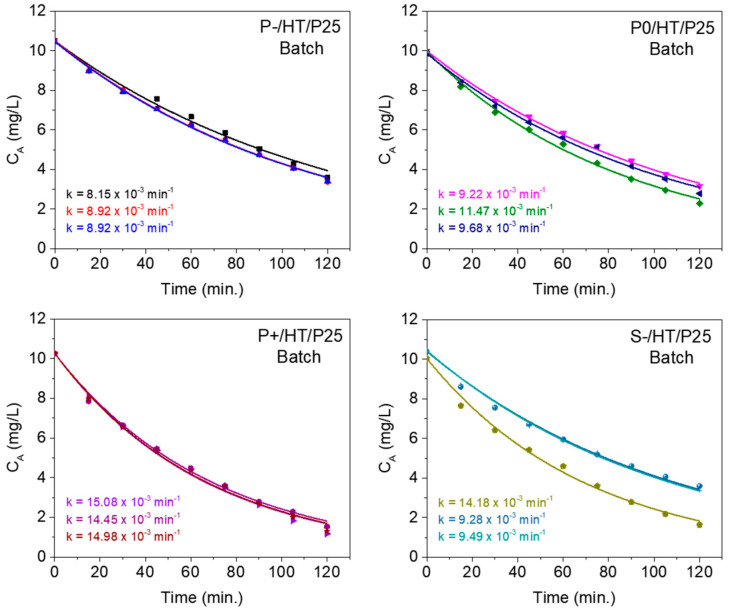
Adjusted kinetic models in batch using the photocatalytic MICROSCAFS^®^ with different porosities and sizes. Kinetic rate constant values (k) are indicated for each case.

The kinetics of MO degradation was assessed using a small laboratory batch reactor, with stirring, in which the approximation to perfect mixing inside the reactor is considered very reasonable. The MO concentration evolution with the reaction time (t) is fitted by Equation (1), stated in the Section 3. The results of the corresponding adjustments are shown in Figure 5, where the quality of all the fittings reinforces the fact that the degradation of MO occurs according to a first-order reaction (A → B). 

It is worth noting that the P+/HT/P25 sample shows the highest reproducibility among all the photocatalytic MICROSCAFS^®^. This is possibly due to the large pore size, which possibly enabled well-distributed and dispersed photocatalytic NPs and ensured a good flow of the pollutant solution, increasing the contact between active sites and pollutant species and avoiding the formation of concentration gradients.

Regarding the continuous flow setup, and taking into account the considerations well-described in the Section 3, the modeling and fitting of the MO concentration’s evolution with the reaction time (t) was carried-out, using Equations (2) and (3).

**Figure 6:**
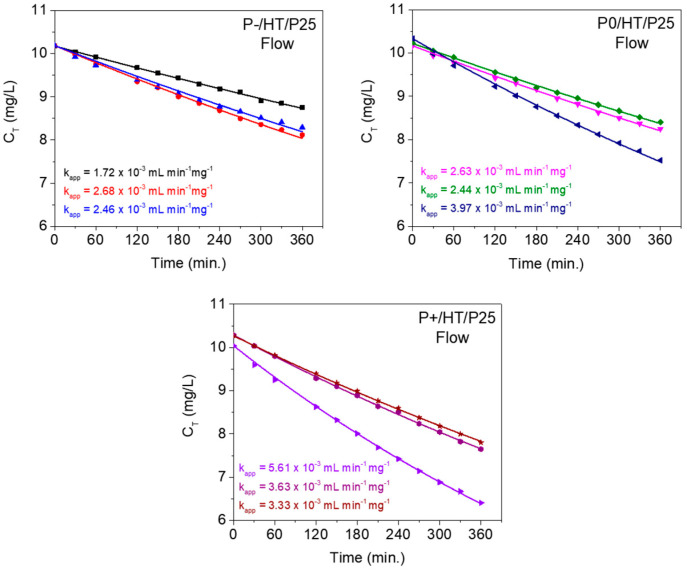
Adjusted kinetic models of the flow reactor using the photocatalytic MICROSCAFS^®^ with different porosities, and a constant tank volume of 200 mL. Apparent flow (adjusted) kinetic rate constant (k_app_) values are indicated for each case.

The best adjusted models of the flow reactor are displayed in Figure 6 in triplicate for the photocatalytic MICROSCAFS^®^ with different porosities.

Table 1 lists the average kinetic rate constant values obtained from experiments in batch conditions, as well as the average apparent flow (adjusted) kinetic rate constant values obtained from experiments in flow conditions for the photocatalysts under study. The constant obtained from the batch reaction studies (min^−1^) was multiplied by the batch reactor volume (50 mL) and divided by the mass of active phase to be comparable with the apparent flow kinetic rate constants (expressed in mL min^−1^ mg^−1^). In this way, the kinetic constants better characterize the activity of the catalyst.

The MO degradation graphs from the flow reactor modeling (Figure 6) clearly show that the adjusted model is able to describe very well the experimental data during the timeframe of the experiment (6 h of reaction). In this case, the fitting process shows that the apparent velocity constant values, k_app_, are lower than those (k) obtained at the batch experiment (Table 1), which is also in agreement with Figure 3b and Figure 4a.

Since the reaction in this study is a photocatalytic heterogeneous transformation, the reactant has to be adsorbed onto the active sites of the solid surface of the photocatalyst, and, simultaneously, the available light and effective irradiance at the solid surface is also essential for the reaction to proceed. There is probably a lower efficiency of mass transfer of the reactant from the fluid to the solid surface and a less favored interaction of the radiation with the active sites of the catalyst in the continuous flow setup when compared to the batch setup. The photocatalytic MICROSCAFS^®^ are densely packed inside the flow reactor chamber, whereas, in batch, they are free-flowing and well dispersed, facilitating their exposure to solar radiation. Also, the surface area exposed to the simulated solar light is much less than in the batch setup. Thus, despite the benefit of the flow setup for real-life applications, the observed reaction rate for the photocatalytic heterogeneous transformation is expected to be reduced when compared to the batch setup. A design optimization of the reactor targeting a larger surface area of exposure to solar light might approximate the flow reactor performance to that of the batch reactor.

**Figure 7:**
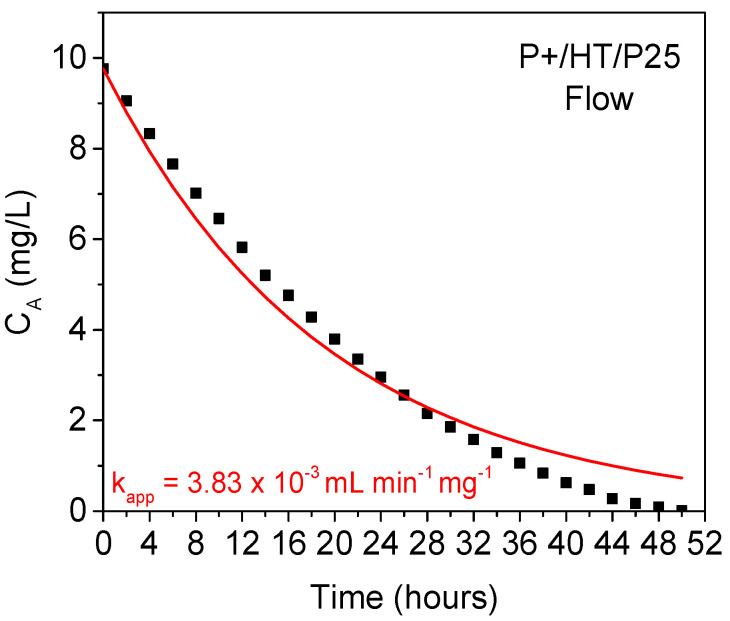
Adjusted kinetic model of the complete MO degradation using the continuous flow reactor.

A full degradation of the MO species present in solution was achieved for ca. 50 h using our continuous flow setup, which is slightly less than the model (Figure 7). This fact suggests that other effects not considered in the model might become relevant, particularly the effect of the MO solution volume, which is made to decrease along the experiment, due to removing aliquots, to ca. 13% of the initial volume, and possibly some evaporation. Additionally, the circular shape of the reactor chamber might be less favorable than, for instance, the well-known tubular reactors, as tubular reactors promote better contact between the solid photocatalyst and liquid pollutant solution. Therefore, the probability for concentration gradients’ generation inside our flow reactor chamber might be considerable. For longer reaction times, these effects might be significant, having a cumulative effect.

Supplementary Table S4 compiles various supported photocatalyst systems in the literature and the corresponding rate constant values factorized to the reactor volume and mass of the active phase. The relevant experimental conditions are described as well. We took into consideration only the literature works where the photocatalytic MO degradation was carried out using supported TiO_2_ NPs [6,7,8,9,10,11,12,35]. Comparing our results with those found in the literature, we found that the k_app_ achieved in the current work for continuous flow experiments was higher than that achieved for SiO_2_ MICROSCAFS^®^ ((4.19 ± 0.7) × 10^−3^ vs. 1.36 × 10^−3^) [7], even for a smaller photocatalyst/pollutant mass ratio (23 vs. 33). It is suggested that the small extra amount of TiO_2_ already present in the SiO_2_-TiO_2_ MICROSCAFS^®^ of the current work might contribute to a more efficient photocatalytic process, as well as the larger interconnected macropores, which have a size distribution that peaked at ~1.95 µm (almost 20 times higher than the reported for SiO_2_ MICROSCAFS^®^). Also, as an extra variable, SiO_2_ MICROSCAFS^®^ were loaded with synthesized TiO_2_ NPs (anatase) and not P25 TiO_2_ NPs, contrary to SiO_2_-TiO_2_ MICROSCAFS^®^, where P25 TiO_2_ NPs were employed. Ljubas et al. reported higher photocatalytic activity achieved by P25 TiO_2_ than by anatase [36]. Compared to a work which used a similar continuous flow reactor and cellulose nanofibrils supports [9], the MICROSCAFS^®^ are still behind, possibly because of their lower transparency to the UV-Vis radiation. However, in regard to chemical and mechanical resistance and durability, MICROSCAFS^®^, due to their inorganic nature, are expected to display a better performance. 

Notably, the present work fits the experimental photocatalytic behavior in flow using the equations for the specific employed continuous flow setup, which is a more accurate methodology to calculate k_app_ than using a batch reactor as a model. Another best practice of the present work is the type of solar source and conditions employed. The 1 sun AM 1.5G reference spectrum was used as the standard for the photocatalytic tests in our work. Also, the full experimental conditions under which the photocatalytic tests were carried out were revealed, with special attention paid to the active area of the device; the calibration protocol; and the properties of the illumination, including spectral irradiance and intensity. Other works herein referred for comparison used unfiltered Hg lamps or even UV lamps, not mimicking sunlight, which does not really comply with the critical need of using solar light in a real application, to minimize energy consumption. Some other works [8,35] used Xe lamps without filters, which emit more UV radiation than the present work lamp, which might explain the higher degradations and photocatalytic activities reported for TiO_2_ NPs. P25 TiO_2_, when immobilized on a PVA film [11], has demonstrated higher activity than the present work; however, the photocatalyst/pollutant mass ratio was three times higher, a UV radiation source was used, and the pH value was not disclosed, which is known to greatly affect the photocatalysis activity, with acidic media being more favorable in this case. For instance, References [6,8,10,12,35], where a batch setup was employed, did not discuss the experimental conditions, such as the irradiance, which is crucial to compare results. Nevertheless, it is worth noting that the present work still yielded a higher reaction rate constant than some UV-irradiated batch systems [10,12]. Systematic computational investigations on TiO_2_ NPs [37] revealed that thermal annealing leads to amorphization (disorder) of the anatase NPs’ surface, inducing valence band edge broadening and higher photoactivities. In our work, besides thermal annealing at 500 °C, P25 TiO_2_ NPs are grafted to the silica–titania MICROSCAFS^®^ and, therefore, are in very close contact with amorphous, low-coordination titania species that exist in the MICROSCAFS^®^. This, together with the wide and interconnected macropore characteristic of the MICROSCAFS^®^, might suggest a reason for the relevant photocatalytic MICROSCAFS^®^ activity achieved in the present work when exposed to solar radiation, which includes only a very small fraction of UV radiation.

### 2.4. Study of the By-Products from Photocatalytic MO Degradation 

**Figure 8:**
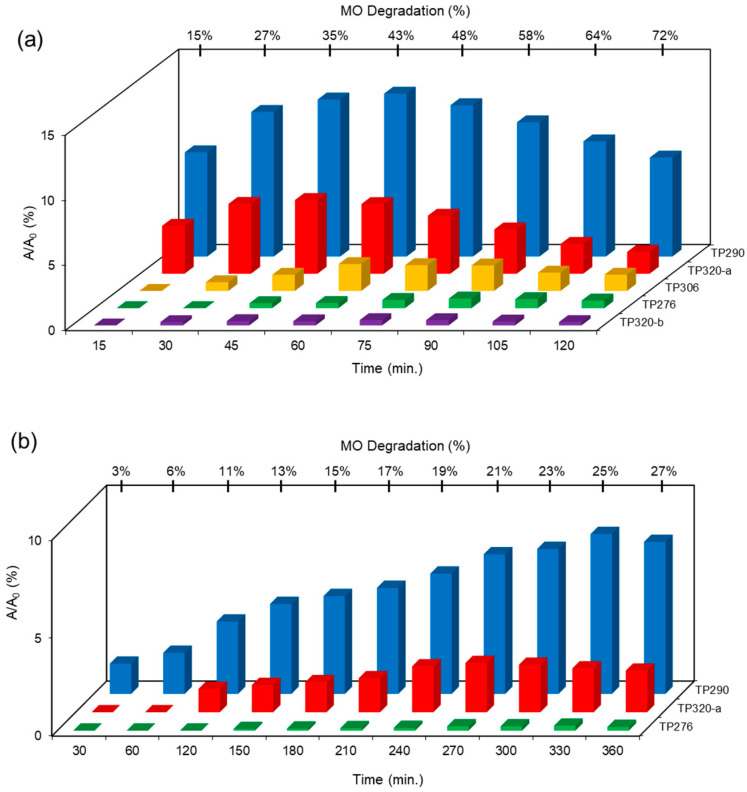
TPs identification of MO photocatalytic degradation during 120 and 360 min, using P0/HT/P25 MICROSCAFS^®^ in the (**a**) batch and (**b**) flow reactor, respectively. A_0_ is the initial MO peak area, obtained by LC-HRMS/MS. The MO degradation values (%) indicated at the top of the graphs were obtained by UV-Vis spectroscopy.

The transformation products (TPs) formed during the photocatalytic degradation of MO using sample P0/HT/P25 were analyzed by LC-HRMS/MS. Figure 8 shows the formation of MO TPs over irradiation time in the presence of the photocatalytic MICROSCAFS^®^ P0/HT/P25, using a batch (Figure 8a) and flow (Figure 8b) reactor. The analysis was carried out up to a solar exposure time of 120 min (batch) and 360 min (continuous flow), which corresponds to a partial MO degradation at 72% and 27%, respectively (by UV-Vis). 

Five by-products were identified in total, whose chemical structure is included in Table 2 in agreement with a degradation mechanism proposed in the literature [38,39,40,41]. More specifically, five by-products were found using the batch reactor, where a more extended MO degradation process was achieved, and three of them using the flow reactor. The proposed ionic formulas for the TPs (Table 2) were supported by tandem mass spectrometry experiments, and the fragmentation pathways for the precursor ions of MO and their TPs are presented in Supplementary Figures S11–S16. TP320-a and TP320-b are isomers, although showing different fragmentation patterns (Supplementary Figures S12 and S16). These isomers are formed because the MO molecular structure contains two benzene groups, and the addition of a hydroxyl radical can occur in two positions. 

Figure 8a shows that MO degradation (in the batch reactor) starts with its partial conversion to the compounds TP290 and TP320-a, following a similar pattern to a previously reported one [29], whose amount starts to decrease after around 60 min of exposure to simulated solar light. The formation of these by-products, TP320-a and TP290, occurs due to the addition of a hydroxyl radical at a benzene group of MO and to the cleavage of a methyl in the dimethylamine group of MO, respectively. After 30 min of irradiation, the formation of the compounds TP306 and TP320-b was detected, due to the cleavage of a methyl in the dimethylamine group of TP320-a and the isomerization of TP320-a, respectively. In the meantime, the loss of both methyl units of the dimethylamine group of MO occurred, and the formation of the compound TP276 was observed. It should be noted that the formation of the compounds TP320-b and TP276 was in a very small amount. Although it was not possible to remove all the TPs, a decrease was observed after around 60 min of irradiation. After 120 min of irradiation, we still identified residual MO species, which were expected because of the incomplete MO degradation at this stage, together with the just-mentioned five TPs in solution. Therefore, a longer irradiation time will be necessary to achieve the complete degradation of both the MO and by-products. 

As for the flow reactor results (Figure 8b), it should be noted that only the initial stages of MO degradation are being analyzed, i.e., until 27% MO degradation. Indeed, MO degradation was found to be slower than in batch conditions, which might explain the lower number of TPs (TP290, TP320-a, and TP276) identified in this case. So, it is found to begin with MO’s conversion to compound TP290, followed by the formation of compound TP320-a after 120 min of irradiation. After 150 min of irradiation, the compound TP276 appears in a very small amount. After 360 min of irradiation, which corresponds to 27% MO degradation (by UV-Vis), we still have TPs and MO species in solution, as expected, suggesting that a longer irradiation period is required for the complete degradation of MO. Comparing the same level of MO degradation at 27%, determined by UV-Vis spectroscopy, i.e., 30 min (batch) and 360 min (flow), a similar trend is observed, with the same TPs being identified, except for TP306 (that appears just in batch).

Finally, regarding the toxicity of the TPs, an Ecological Structure–Activity Relationships (ECOSAR) prediction was carried out, and the results are presented in Table 3 [42]. As can be seen, all the TPs are harmless for fish and for green algae (except TP276) in terms of acute toxicity. Regarding chronic toxicity for fish species, TP290 remains harmless; TP320-a, TP320-b, and TP306 are harmful; and the by-product found in residual quantity (TP276) is toxic. For green algae, TP320-b, TP306, and TP290 are harmless, while the remaining TPs are harmful, as MO is. It should be stressed that the by-product found in a higher amount, TP290, is a non-toxic (harmless) by-product for fish, daphnid, and green algae, in what regards both acute and chronic toxicity.

### 2.5. Scavenger’s Study on the Photocatalytic MICROSCAFS^®^ and Photocatalysis Mechanistic Study 

**Figure 9:**
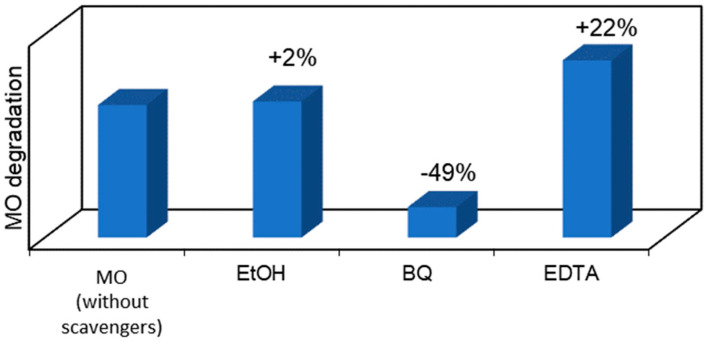
MO degradation evolution after 120 min of solar irradiation using photocatalytic MICROSCAFS^®^ P+/HT/P25 when in the presence of selected scavengers: EtOH, BQ, and EDTA to quench ^•^OH, ^•^O_2_^−^, and h+, respectively.

To go further in this study and to analyze whether the MO photocatalytic degradation takes place via oxygen radical species, such as ^•^O_2_^−^, ^•^OH or via direct positive holes (h^+^) or electron (e^−^) transfer, radical scavengers were added to the MO aqueous solution to remove the corresponding reactive species and compared with the photocatalytic reaction without scavenger (Figure 9). In particular, BQ, EtOH, and EDTA were used as ^•^O_2_^−^, ^•^OH, and h^+^ scavengers, respectively. Considering the photocatalytic performance results discussed above, the P+/HT/P25 sample was chosen for this study. The obtained results revealed that when using EtOH, an ^•^OH scavenger, no significant differences in the pollutant removal were observed. On the other hand, the most pronounced photodegradation suppression can be seen when BQ was added to the MO solution. The addition of this well-known ^•^O_2_^−^ scavenger, during MO photocatalytic degradation, reduced by around 49% the MO removal (absolute value). On the other hand, the degradation of MO was enhanced in the presence of EDTA. When this h^+^ scavenger was added to the system, an increase of 22% (absolute value) in the MO removal was obtained comparing to the case without scavengers. Similar results were already published by other authors, such as Liu et al. [43], who used EDTA as a scavenger in the degradation of dyes. This obtained result can be justified because EDTA acts both as an h^+^ scavenger and also an e^−^ donor [44]. In this sense, the recombination of the e−/h+ was reduced, since the EDTA was used as a scavenger to quench h^+^, and, consequently, more e^−^ can react with the O_2_ present at the surface to produce ^•^O_2_^−^. Furthermore, we could see that the superoxide radicals, ^•^O_2_^−^, are the main oxidant species involved in this photocatalytic process. Therefore, in the presence of EDTA, we have more e^−^ in the system that is able to provide O_2_/O_2_^•−^ reduction in the conduction band (CB), thus enhancing the MO degradation under solar irradiation. Therefore, we can conclude that MO degradation does not take place directly through ^•^OH and h^+^, but through ^•^O_2_^−^, which are the main oxidant species involved in this photocatalytic process.

**Figure 10:**
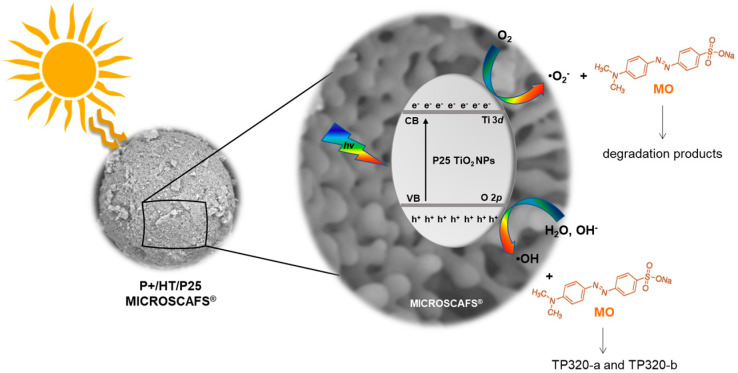
The photoactivation mechanism proposed for photocatalytic MICROSCAFS^®^ P+/HT/P25, used as a catalyst for the photodegradation of MO under solar light irradiation. Photocatalytic MICROSCAFS^®^, P+/HT/P25, are composed of MICROSCAFS^®^ P+/HT with immobilized photocatalytic P25 NPs.

A mechanism for the solar light activation of the P+/HT/P25 MICROSCAFS^®^ is proposed in Figure 10. When the MICROSCAFS^®^ are exposed to solar radiation with energy enough to promote the photogeneration of charge carriers, e^−^ and h^+^ will be generated in the CB and valence band (VB), respectively. The excited electrons in the CB will react with the adsorbed O_2_ and reduce them into ^•^O_2_^−^. This superoxide radical plays an active role in the photodegradation of MO, as concluded by the scavenger’s study, and also contributes to the longer lifetime of the charge carriers. Furthermore, the ^•^O_2_^−^ can also produce H_2_O_2_ derived from the O_2_ photo-reduction that also enhances the photodegradation of MO. On the other hand, in the VB, the photogenerated h^+^ reacts with either the adsorbed H_2_O or OH^−^, resulting in the formation of ^•^OH radicals. These reactive species do not play an active role in this photodegradation system; however, they are responsible for the formation of two TPs detected by HRMS, the isomers TP320-a and TP320-b, formed due to the hydroxylation of MO. In our previous work, we also observed a hydroxylation of the antidepressant amitriptyline forming three isomers during their photocatalytic degradation due to the addition of hydroxyl radicals in different positions on the amitriptyline molecule [45]. Similar results were also published during the photodegradation of several compounds, such as sulfaclozine, sulfonamides, and sulfachloropyridazine, reporting that the photocatalytic degradation of the studied pollutants starts with their hydroxylation, due to the addition of ^•^OH to the aniline ring [42,46,47,48].

## 3. Materials and Methods

### 3.1. Materials

Tetraethyl orthosilicate (TEOS, 98%, Sigma-Aldrich, Burlington, MA, USA), (3-glycidyloxypropyl) trimethoxy silane (GPTMS, Xiameter OFS-6040, >98.5%, kindly supplied by Dow, MI, USA), titanium (IV) isopropoxide (TiPOT, 98%, Acros Organics, ThermoFisher Scientific, Waltham, MA, USA), glacial acetic acid (≥99%, Fisher Chemical, ThermoFisher Scientific, Waltham, MA, USA), decahydronaphthalene (decalin, mixture of cis and trans isomers, 98%, Merck, Darmstadt, Germany), sorbitan monooleate nonionic biodegradable surfactant (Span^®^ 80, HLB: 4.3, Merck, Darmstadt, Germany), Pluronic^®^ P-123 nonionic surfactant, a copolymer comprising poly(ethylene oxide) (PEO) and poly(propylene oxide) (PPO) in an alternating linear fashion (HLB: 8, Sigma-Aldrich, St. Louis, MO, USA), ammonia 25% aqueous solution (Chem-Lab, Zedelgem, Belgium), Aeroxide^®^ P25 Degussa TiO_2_ NPs (Acros Organics, ThermoFisher Scientific, Waltham, MA, USA), methyl orange (MO, Fisher Chemical, ThermoFisher Scientific, Waltham, MA, USA), p-benzoquinone (BQ, Merck, Darmstadt, Germany), ethylenediaminetetraacetic acid disodium salt dihydrate (EDTA, Panreac, Barcelona, Spain), and ethanol (EtOH, ≥99.8%, Fisher Chemical, ThermoFisher Scientific, Waltham, MA, USA).

### 3.2. Synthesis of the MICROSCAFS^®^

Silica–titania (ST) MICROSCAFS^®^ were synthesized using the same method recently published by our research group [27,49]. It consisted of two main steps: step 1—silane precursors’ hydrolysis and simultaneous complexation of the titania precursor; and step 2—alkaline condensation in the water droplets of an emulsion medium and MICROSCAFS^®^’ formation. The synthesis process is described in detail in the Supplementary Materials.

Four different MICROSCAFS^®^ were studied in this work. Sample “P0” followed the standard procedure reported in the Supplementary Materials and was considered to be the reference in this paper. Samples “P−” and “P+” were synthesized using a lower and higher volume of GPTMS than the reference, respectively. Sample “S−” was synthesized using a higher volume of Span^®^ 80, together with another surfactant, Pluronic^®^ P-123, both added to the water phase of the emulsion. Table 4 lists the four different samples of study and corresponding variable synthesis parameters.

### 3.3. Preparation of the Photocatalytic MICROSCAFS^®^

All the four samples of MICROSCAFS^®^ were used as a scaffolding or support to commercial Aeroxide^®^ P25 Degussa TiO_2_ NPs, using a wet impregnation method adapted from [7], forming the photocatalytic MICROSCAFS^®^. The wet impregnation method is described in detail in the Supplementary Materials. The acronyms “HT” and “P25” represent samples that were heat-treated and impregnated with P25 NPs, respectively.

### 3.4. Characterization 

The morphology and the elemental composition of the MICROSCAFS^®^ were evaluated through scanning electron microscopy (SEM) images and energy-dispersive X-ray spectroscopy (EDS) data acquired using a Phenom ProX G6 benchtop SEM (ThermoScientific, Waltham, MA, USA). Internal porosity was observed on purposedly broken particles. Prior to observation, a 15 nm gold–palladium layer was sputtered onto the samples, using a turbomolecular pumped coater Q150T ES (Quorum Technologies, Lewes, UK). The MICROSCAFS^®^ diameter was measured manually using the ImageJ software, as previously reported [27]. The transmission electron microscopy (TEM) images of the P25 TiO_2_ NPs (shown in Supplementary Materials) were obtained using a H8100 microscope (Hitachi, Tokyo, Japan) operating at 300 kV. 

Dried, P25 TiO_2_ NP-loaded, and -unloaded MICROSCAFS^®^’ porosity was assessed by mercury intrusion porosimetry (MIP) using an Autopore IV 9500 Mercury Porosimeter (Micromeritics Instrument Corporation, Norcross, GA, USA) and N_2_ adsorption/desorption isotherms using an ASAP 2010 adsorption analyzer (Micromeritics, Instrument Corporation, Norcross, GA, USA), in the same fashion as in [27]. 

The chemical structure of the samples was assessed through Fourier-transform infrared spectroscopy (FTIR), using a Spectrum Two spectrometer (PerkinElmer, Waltham, MA, USA) coupled with a universal attenuated total reflectance (ATR) accessory (PerkinElmer, Waltham, MA, USA). The spectra were obtained at 4 cm^−1^ resolution, with 8 scans of data accumulation.

The UV-Vis diffuse-reflectance spectra (UV-Vis DRS) were measured by a V-750 UV-Vis spectrophotometer (JASCO Corporation, Tokyo, Japan) equipped with an integrating sphere and converted into absorption units (F_KM_), using the Kubelka–Munk function, which is related to the diffuse reflectance by the expression F_KM_(R) = (1 − R)^2^/2R [50,51,52].

### 3.5. Photocatalytic Tests

The photocatalytic MICROSCAFS^®^ were tested in a batch reactor and, as a proof of concept for continuous transformation processes, also in a continuous flow reactor. The batch reactor was a 100 mL jacketed vessel connected to a water recirculatory bath at 19 °C. In each batch test, 50 mg of photocatalytic MICROSCAFS^®^ was dispersed in 50 mL of a MO aqueous solution with an initial MO concentration of 10 mg/L and pH of 7, under magnetic stirring. The system was kept in the darkness for 1 h to study possible adsorption, followed by illumination during 2 h with a solar lamp. For this purpose, a Newport 94011A-ES solar simulator (Newport Corporation, Irvine, CA, USA) was employed, with a 100 W Xe lamp with reflector (Newport Corporation, Irvine, CA, USA) that produces a 3.8 cm × 3.8 cm collimated beam. This solar simulator includes an AM1.5G air mass filter, which provides a Class A spectral performance based on current applicable standards. The solar lamp’s distance from the solution’s surface was adjusted to correspond to the irradiance of 1 sun (1000 W/m^2^). The simulated solar light enters the reactor through a quartz window of 4 cm diameter (12.57 cm^2^) placed at the top of the reactor. The transmission spectrum of the quartz window is shown in Supplementary Figure S1. It transmits at ca. 92% from 380 to 900 nm. Aliquots of 1 mL were taken every 20 min during the dark phase and every 15 min during the light phase and centrifuged. The MO solution concentration at each specific time was then determined by measuring the absorbance at 464 nm, using a JASCO V-750 UV-Vis spectrophotometer (JASCO Corporation, Tokyo, Japan). All the experiments were conducted for three replicates at 19 °C, and the mean results were reported.

Additionally, photocatalytic experiments (in batch) were carried out using distinct radical scavengers. EtOH was employed as ^•^OH scavenger in the photocatalysis reaction, EDTA was chosen as h^+^ quencher, and BQ was added to the system as ^•^O_2_^−^ scavenger. The MO photocatalytic experiments were performed using the same conditions described above, while using separately 0.5 mM of each scavenger. 

The flow tests were carried out using a setup built to allow a continuous flow with recirculation to an intercalated tank (Supplementary Figure S2). The photoreactor employed was adapted from the one previously reported [7,9]. This reactor comprises three separate acrylic pieces that, when assembled, form a chamber (solar reactor) where the photocatalytic MICROSCAFS^®^ sample can be placed, and the solution can circulate throughout the sample. The top of this chamber displays a quartz window of 2 cm in diameter (3.14 cm^2^), and the transmission spectrum is shown in Supplementary Figure S1, on which the solar light will impinge, and the back contains the inlet and outlet of the circulating solution. In total, 200 mg of photocatalytic MICROSCAFS^®^ was inserted and compressed inside this chamber, around ~1.5 mm thick, resulting in a chamber volume of 0.47 cm^3^. We placed a filter paper (1318 from FILTER-LAB^®^, Barcelona, Spain) at the back of the chamber, where the inlet and outlet of the circulating solution were placed, to prevent the MICROSCAFS^®^ from exiting the chamber. The reactor was connected to a tank with two 78 cm long TYGON^®^ hoses (Hirschmann Laborgeräte, Eberstadt, Germany), and 200 mL of MO solution (10 mg/L) was made to circulate at a constant flow at 10 mL/min, with the help of a Rotarus^®^ standard 50 peristaltic pump (Hirschmann Laborgeräte, Eberstadt, Germany) during the tests. The reactor was exposed to a constant 1000 W/m^2^ (1 sun) at the exterior surface of the quartz window over 6 h, after 1 h of darkness, to account for any possible adsorption. It should be noted that, at the inner surface of the quartz window, the irradiance was measured and found to be 980 W/m^2^, which is not a significant difference. The concentration of the pollutant was checked every 30 min by measuring the absorbance at 464 nm of 1 mL MO aq. solution aliquots. The same solar simulator and reference solar cell from the batch tests were used. All the experiments were conducted for three replicates at 25 °C, and the mean results were reported.

In the stability (recyclability) tests, six consecutive cycles were performed by simply replacing, in the stirred tank, the degraded MO solution by a new one at 10 mg/L, and the corresponding MO degradation was recorded to examine the stability and reusability of the photocatalytic MICROSCAFS^®^ at a catalyst/pollutant mass ratio of 23, pH of 7, under solar light (1 sun) exposure and 25 °C.

### 3.6. Kinetics Modeling of the Photocatalytic Performance 

The kinetics for MO photocatalytic degradation was analyzed in batch and flow conditions. By assuming that it follows a first-order transformation, with the reaction rate given by r = $kC_{A}$, the MO concentration evolution with the reaction time (t) can be described by Equation (1):

(1) $$C_{A}=C_{A_{0}}e^{-kt}$$

where k is the kinetic rate constant, $C_{A}$ is the MO concentration inside the reactor at time t under light irradiation, and $C_{A_{0}}$ is the initial MO concentration before irradiation. The batch reactor is known to allow a good efficiency of mass transfer from the fluid to the surface of the solid photocatalyst, where the transformation takes place; for heterogeneous catalysis to occur, the MO pollutant dissolved in the liquid medium must contact and be adsorbed on the active sites of the photocatalyst. 

The constant *k* was determined by fitting Equation (1) to the experimental points of *C_A_* as a function of time, using the sum of the square’s deviations minimization method. 

**Figure 11:**
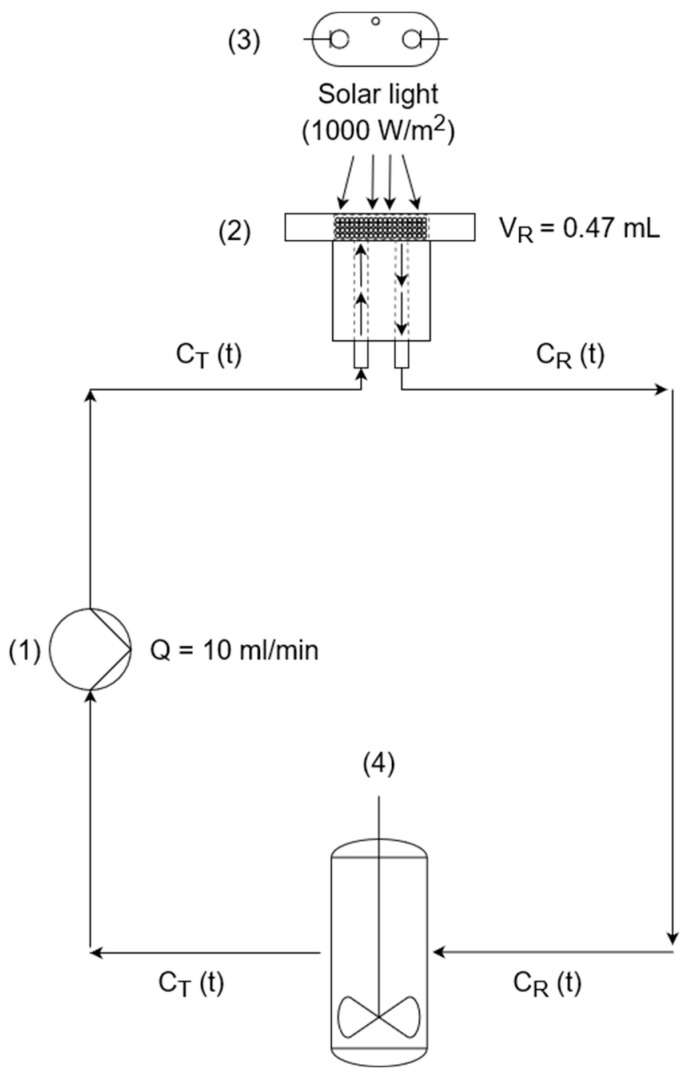
Process flow diagram of the flow reactor: (1) peristaltic pump; (2) continuous flow chamber (solar reactor, side view) containing the photocatalytic MICROSCAFS^®^; (3) solar simulator (Xe lamp), and (4) stirred tank.

To model the experimental MO photodegradation system using our continuous flow setup (continuous flow with recirculation to an intercalated tank), the following process flow diagram, shown in Figure 11, was employed, exhibiting the small continuous flow chamber (solar reactor) (2) with complete recirculation. 

The reactor, exposed to the solar simulator (3), is connected to the stirred tank (4) with 200 mL of volume in a closed-circuit pumped by a peristaltic pump (1) at a constant volumetric flow rate of 10 mL/min.

Homogeneous conditions were considered inside the solar reactor (chamber), which is a reasonable approximation when the conversion of the reactant for each pass of a volume element of fluid through the reactor is low. The inlet concentration of the solar reactor is the same as the outlet concentration of the tank ($C_{T}\left(t\right)$), while the inlet concentration of the tank is equal to the outlet concentration of the solar reactor ($C_{R}\left(t\right)$), both changing over time. 

After the peristaltic pump starts, the MO solution enters the reactor and flows throughout the photocatalytic MICROSCAFS^®^ interparticle spaces and internal pores. We consider that the flow solar reactor works in differential conditions, at constant flow and almost negligible concentration gradient inside, since the pollutant solution quickly flows through it (~2.8 s as mean residence time). The pollutant fluid stream leaving the reactor is mixed with the solution that remains behind in the dark, in the stirred tank (Figure 11). 

The mass balance equations describing the transient behavior of the reactor and of the tank where no reaction occurs are given by Equations (2) and (3), respectively.

(2) $$V_{R}\frac{dC_{R}\left(t\right)}{dt}=QC_{T}\left(t\right)-QC_{R}\left(t\right)-kC_{R}\left(t\right)W$$

(3) $$V_{T}\frac{dC_{T}\left(t\right)}{dt}=QC_{R}\left(t\right)-QC_{T}\left(t\right)$$

where $V_{R}$ is the volume of the flow reactor in L, $V_{T}$ is the tank volume in L, Q is the volumetric flow in L/min, $C_{R}\left(t\right)$ is the MO concentration in the reactor (mg/L), $C_{T}\left(t\right)$ is the MO concentration in the tank (mg/L), and W is the mass of the photocatalytic MICROSCAFS^®^ placed inside the reactor (200 mg). The kinetic rate constant, k, is the variable parameter of this mathematical model. 

The simultaneous resolution (integration) of Equations (2) and (3) was accomplished using the Euler method, allowing for the determination of the evolution of $C_{R}\left(t\right)$ and $C_{T}\left(t\right)$. Several integrations were carried out until the best value of k is reached (k_app_), yielding the lowest sum of the squared deviations between the experimental and model $C_{T}\left(t\right)$ values.

### 3.7. Analytical Methods for Photocatalysis By-Products Determination

The transformation products (TPs) formed during the photocatalytic degradation of MO were analyzed by LC-HRMS/MS. The MO solutions were analyzed on an UHPLC Elute system interfaced with a QqTOF Impact II mass spectrometer equipped with an ESI source, operating in the negative mode (Bruker, Billerica, MA, Germany). Chromatographic separation was carried out under gradient conditions, using a RF-C18 Kinetex column 100 Å (150 mm × 2.1 mm, 2.6 μm particle size, Phenomenex, Torrance, CA, EUA). Detailed settings on LC-HRMS/MS settings were described in our previous work [29]. The TPs peak areas (A) were normalized with respect to MO at t = 0 min (A/$A_{0}$), and their variation as a function of irradiation time is shown in 3D bar graphs. The TPs are identified by HRMS based on their accurate *m*/*z* values released as deprotonated molecules ([M − H]^−^), considering the accuracy and precision of the measurement parameters, such as error (ppm) and mSigma. The molecular formulas were validated by extracting the ionic chromatograms from the raw data, and accurate mass isotopic patterns and fragmentation paths were evaluated, supporting the respective proposed chemical structures. Detailed equipment specifications and experimental protocol for calibration, data acquisition, and processing are described elsewhere [53]. 

Lastly, the toxicity of the TPs was assessed by in silico predictions using the free available tool Ecological Structure Activity Relationship (ECOSAR v1.11) to estimate ecotoxicity. From the ECOSAR prediction of toxicity, three different organisms were selected, fish, daphnid, and green algae, for the acute and chronic ecotoxicity. Detailed specifications on toxicity prediction by ECOSAR are described in our previous work [54] for a different system and other chemical pollutants.

## 4. Conclusions

Silica–titania MICROSCAFS^®^ with pore sizes at different scales, and different particle size diameters were successfully developed with high reproducibility using an adapted sol–gel method, involving polymerization-induced phase separation. They were loaded with commercial photocatalytic P25 TiO_2_ NPs, whose immobilization within the MICROSCAFS^®^ was promoted by HT at 500 °C. MICROSCAFS^®^ characteristics, mainly macropore size, directly affected the photocatalytic performance in what regards the organic dye MO degradation. MICROSCAFS^®^ with greater interconnected pore sizes exhibited higher photocatalytic activity both in batch and in flow setups. Their engineered macroporous network favors mass transport, and therefore the flow of the pollutant solution throughout the pores confers higher accessibility of the pollutant molecules to the active sites and provides conditions to spread the photons’ pathway inside the pore channels. The size of the particles appears not to have such a relevant effect on the photocatalytic performance. MICROSCAFS^®^ are herein shown to be a viable solution for environmental remediation (e.g., wastewater purification), using straightforward flow conditions, and do not require any separation step, like centrifugation or filtration, which otherwise would be very expensive on an industrial scale, or of high complexity in remote areas such as rural villages. Experiments in a flow reactor with total recirculation to an intercalated tank acted as a proof of concept for continuous transformation processes targeting real-life applications. A modeling study of our flow setup was developed and tested, which yields more accurate results and successfully predicts the reaction evolution. There are other works from the literature that have reported higher degradation rates than the present work; however, the employed photocatalyst/pollutant ratio was generally higher, and/or they used UV light (instead of solar light) which gives rise to high photo-efficiency, or relevant information on the experimental conditions was missing. Our process used solar light as the light source, which has only ~5% UV. Finally, the design of a new larger scale flow reactor with higher photocatalyst/pollutant/light contact is promising to further enhance photocatalytic activity, allowing for the application of this system on an industrial scale, providing a viable solution for the global water pollution problem.

## Figures and Tables

**Table 1:** Average kinetic rate constants (batch) and apparent flow reactor rate constants of the photocatalytic MICROSCAFS^®^.

Sample Acronym	Average Kinetic Rate Constant k (mL min^−1^ mg^−1^)	Average Apparent Flow Reaction Rate Constant k_app_ (mL min^−1^ mg^−1^)
P−/HT/P25	(3.47 ± 0.1) × 10^−2^	(2.28 ± 0.3) × 10^−3^
P0/HT/P25	(4.40 ± 0.3) × 10^−2^	(3.01 ± 0.5) × 10^−3^
P+/HT/P25	(6.45 ± 0.08) × 10^−2^	(4.19 ± 0.7) × 10^−3^
S−/HT/P25	(4.99 ± 0.7) × 10^−2^	n. a.

n. a.—not available.

**Table 2:** LC-HRMS/MS identification of MO and their degradation by-products (TPs).

Compound	Structure	t*_R_* (min)	Proposed Empirical Formula	[M−H]^−^ [*m/z* (∆ppm) mSigma]
**MO**	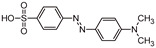	9.5	C_14_H_15_N_3_O_3_S	[304.0770 (−2.9; 6.5)]
**TP320-a**	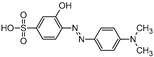	10.9	C_14_H_15_N_3_O_4_S	[320.0722 (−3.7; 9.2)]
**TP306**	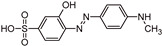	9.0	C_13_H_13_N_3_O_4_S	[306.0562 (−2.7;12.5)]
**TP290**	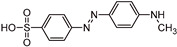	7.8	C_13_H_13_N_3_O_3_S	[290.0614 (−3.2; 10.5)]
**TP276**	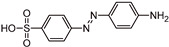	5.8	C_12_H_11_N_3_O_3_S	[276.0453 (−3.2; 21.6)]
**TP320-b**	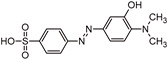	3.9	C_14_H_15_N_3_O_4_S	[320.0712 (−0.2; 9.7)]

**Table 3:** Toxicological assessment of MO and their TPs by ECOSAR.

Compound	Acute Toxicity			Chronic Toxicity		
	(mg/L)			(mg/L)		
	Fish (LC_50_)	Daphnid (LC_50_)	Green Algae (EC_50_)	Fish (C*h*V)	Daphnid (C*h*V)	Green Algae (C*h*V)
**MO**	1513.99	793.35	424.07	134.62	61.88	92.88
**TP320-a**	173.97	39.22	192.16	15.97	7.47	91.02
**TP320-b**	592.29	97.78	512.74	49.07	18.64	245.03
**TP306**	433.60	76.62	395.85	36.73	14.60	188.80
**TP290**	4479.49	2231.63	967.94	375.25	151.23	189.43
**TP276**	922.37	2.07	9.26	5.36	0.13	13.94
Harmless LC50/EC50/ChV > 100		Harmful 100 > LC50/EC50/ChV > 10		Toxic 10 > LC50/EC50/ChV > 1		Very toxic LC50/EC50/ChV < 1

**Table 4:** MICROSCAFS^®^’ synthesis parameters, which suffered variation.

Sample Acronym	Volume of GPTMS (mL)	Volume of Span^®^ 80 (mL)	Mass of Pluronic^®^ P123 (g)
P−	10.4	6.0	0
P0 (reference)	13.8	6.0	0
P+	17.3	6.0	0
S−	13.8	7.7	2.3

## Data Availability

Data available on the Zenodo platform under the following DOI: https://doi.org/10.5281/zenodo.10613584 (accessed on 20 May 2024).

## Supplementary Materials

The following supporting information can be downloaded at https://www.mdpi.com/article/10.3390/ijms25115958/s1.

## References

[1] Lin J., Ye W., Xie M., Seo D.H., Luo J., Wan Y., Van der Bruggen B. (2023). Environmental Impacts and Remediation of Dye-Containing Wastewater. Nat. Rev. Earth Environ..

[2] Saravanan A., Deivayanai V.C., Kumar P.S., Rangasamy G., Hemavathy R.V., Harshana T., Gayathri N., Alagumalai K. (2022). A Detailed Review on Advanced Oxidation Process in Treatment of Wastewater: Mechanism, Challenges and Future Outlook. Chemosphere.

[3] Mancuso A., Iervolino G. (2022). Synthesis and Application of Innovative and Environmentally Friendly Photocatalysts: A Review. Catalysts.

[4] Rauf M.A., Ashraf S.S. (2009). Fundamental Principles and Application of Heterogeneous Photocatalytic Degradation of Dyes in Solution. Chem. Eng. J..

[5] Park S., Choi G.R., Lee J.C., Kim Y.C., Oh D., Cho S., Lee J.-H. (2010). Organic and Inorganic Binder-Coating Properties for Immobilization of Photocatalytic ZnO Nanopowders. Res. Chem. Intermed..

[6] Jiang Y., Liu A. (2023). Cornstalk Biochar-TiO_2_ Composites as Alternative Photocatalyst for Degrading Methyl Orange. Environ. Sci. Pollut. Res..

[7] Marques A.C., Vale M., Vicente D., Schreck M., Tervoort E., Niederberger M. (2021). Porous Silica Microspheres with Immobilized Titania Nanoparticles for In-Flow Solar-Driven Purification of Wastewater. Glob. Chall..

[8] Wu A., Wang D., Wei C., Zhang X., Liu Z., Feng P., Ou X., Qiang Y., Garcia H., Niu J. (2019). A Comparative Photocatalytic Study of TiO_2_ Loaded on Three Natural Clays with Different Morphologies. Appl. Clay Sci..

[9] Lucchini M.A., Lizundia E., Moser S., Niederberger M., Nyström G. (2018). Titania-Cellulose Hybrid Monolith for In-Flow Purification of Water under Solar Illumination. ACS Appl. Mater. Interfaces.

[10] Stewart B.D., Andrews L.G., Pelletier B.S., Daly C.A., Boyd J.E. (2015). Porous PMMA-Titania Composites: A Step towards More Sustainable Photocatalysis. J. Water Process Eng..

[11] Lei P., Wang F., Gao X., Ding Y., Zhang S., Zhao J., Liu S., Yang M. (2012). Immobilization of TiO_2_ Nanoparticles in Polymeric Substrates by Chemical Bonding for Multi-Cycle Photodegradation of Organic Pollutants. J. Hazard. Mater..

[12] Ökte A.N., Karamanis D., Tuncel D. (2014). Dual Functionality of TiO_2_-Flyash Nanocomposites: Water Vapor Adsorption and Photocatalysis. Catal. Today.

[13] da Silva T.C.A., Marchiori L., Mattos B.O., Ullah S., Barud H.d.S., Domeneguetti R.R., Rojas-Mantilla H.D., Zanoni M.V.B., Rodrigues-Filho U.P., Ferreira-Neto E.P. (2023). Designing Highly Photoactive Hybrid Aerogels for In-Flow Photocatalytic Contaminant Removal Using Silica-Coated Bacterial Nanocellulose Supports. ACS Appl. Mater. Interfaces.

[14] Hu Y., Dong H., Tapa A.R., Shamsi J., Shayeh J.S., Trokourey A., Liu B., Zhao X., Xie Y. (2023). NaBr-Assisted Aqueous Synthesis of Perovskite-Embedded PbBr(OH) Hierarchical Nanostructures for Dye Photodegradation. ACS Appl. Nano Mater..

[15] Wu J., Ke K., Qin N., Lin E., Kang Z., Bao D. (2023). Magnetically Retrievable Fe_3_O_4_@SiO_2_@ZnO Piezo-Photocatalyst: Synthesis and Multiple Catalytic Properties. J. Colloid. Interface Sci..

[16] Sun G., Li N., Zuo S., Shen W., Wu M., Li Q., Shi M., Ma J. (2022). Piezo-Photocatalysis over Phase-Engineered MoSe2 Modified Bi2WO6 Hierarchical Microspheres: Utilizing Piezoelectric Effect to Enhance Photocatalytic Performance. Ceram. Int..

[17] Wang J., Sgarzi M., Němečková Z., Henych J., Licciardello N., Cuniberti G. (2022). Reusable and Antibacterial Polymer-Based Nanocomposites for the Adsorption of Dyes and the Visible-Light-Driven Photocatalytic Degradation of Antibiotics. Glob. Chall..

[18] Imran M., Ashraf W., Hafiz A.K., Khanuja M. (2022). Synthesis and Performance Analysis of Photocatalytic Activity of ZnIn_2_S_4_ Microspheres Synthesized Using a Low-Temperature Method. ACS Omega.

[19] Qin M., Jin K., Li X., Wang R., Zhao Y., Wang H. (2022). Bi Nanosphere-Decorated Oxygen-Vacancy BiOBr Hollow Microspheres with Exposed (110) Facets to Enhance the Photocatalytic Performance for the Degradation of Azo Dyes. N. J. Chem..

[20] Kim J., Jo S., Lee W., Lim J., Seung Lee T. (2022). Moving Photocatalyst of a Titanium Dioxide-Based Micromotor Asymmetrically Decorated with Conjugated Polymer Dots. Mater. Des..

[21] Li X., Raza S., Liu C. (2021). Preparation of Titanium Dioxide Modified Biomass Polymer Microspheres for Photocatalytic Degradation of Rhodamine-B Dye and Tetracycline. J. Taiwan Inst. Chem. Eng..

[22] Liang H., Wang S., Lu Y., Ren P., Li G., Yang F., Chen Y. (2020). Highly Efficient and Cheap Treatment of Dye by Graphene-Doped TiO_2_ Microspheres. Water Sci. Technol..

[23] He J., Liu Q., Zhang Y., Zhao X., Zhang G., Xiao B., Fu K. (2023). In Situ Synthesis of the Mesoporous C–TiO_2_ Microspheres Derived from Partial Hydrolysis Tetrabutyl Titanate for Enhanced Photocatalytic Degradation under Visible Light. Mater. Res. Bull..

[24] Feng X., Gu L., Wang N., Pu Q., Liu G. (2023). Fe/N Co-Doped Nano-TiO_2_ Wrapped Mesoporous Carbon Spheres for Synergetically Enhanced Adsorption and Photocatalysis. J. Mater. Sci. Technol..

[25] Ren X., Zhang X., Guo R., Li X., Peng Y., Zhao X., Pu X. (2021). Hollow Mesoporous G-C_3_N_4_/Ag_2_CrO_4_ Photocatalysis with Direct Z-Scheme: Excellent Degradation Performance for Antibiotics and Dyes. Sep. Purif. Technol..

[26] Vale M., Loureiro M.V., Ferreira M.J., Marques A.C. (2020). Silica-Based Microspheres with Interconnected Macroporosity by Phase Separation. J. Solgel Sci. Technol..

[27] Vale M., Orišková S., Mariquito A., Reis L., Pinto M., Marques A.C. (2023). Multicomponent Oxide Microspheres with Designed Macroporosity (MICROSCAFS^®^): A Customized Platform for Chemicals Immobilization. RSC Adv..

[28] Loureiro M.V., Vale M., De Schrijver A., Bordado J.C., Silva E., Marques A.C. (2018). Hybrid Custom-Tailored Sol-Gel Derived Microscaffold for Biocides Immobilization. Microporous Mesoporous Mater..

[29] Fernandes S.M., Barrocas B.T., Nardeli J.V., Montemor M.F., Maçoas E., Oliveira M.C., de Carvalho C.C.C.R., Lauria A., Niederberger M., Marques A.C. (2024). Maximizing Photocatalytic Efficiency with Minimal Amount of Gold: Solar-Driven TiO_2_ Photocatalysis Supported by MICROSCAFS^®^ for Facile Catalyst Recovery. J. Environ. Chem. Eng..

[30] Loureiro M.V., Ciriminna R., Lourenço M.J., Santos L.F., De Schrijver A., Bordado J.C., Pagliaro M., Marques A.C. (2017). Organically-Modified Silica Based Microspheres for Self-Curing Polyurethane One Component Foams. Microporous Mesoporous Mater..

[31] Marques A.C., Loureiro M.V., Lourenço M.J., De Schrijver A., Bordado J.C. (2017). Amino Surface Functionalized Microcapsules as Curing Agents for Polyurethane Foams. Mater. Manuf. Process..

[32] Nagar N., Devra V. (2019). A Kinetic Study on the Degradation and Biodegradability of Silver Nanoparticles Catalyzed Methyl Orange and Textile Effluents. Heliyon.

[33] Benz D., Van Bui H., Hintzen H.T., Kreutzer M.T., van Ommen J.R. (2022). Mechanistic Insight into the Improved Photocatalytic Degradation of Dyes for an Ultrathin Coating of SiO_2_ on TiO_2_ (P25) Nanoparticles. Chem. Eng. J. Adv..

[34] Luna A.L., Matter F., Schreck M., Wohlwend J., Tervoort E., Colbeau-Justin C., Niederberger M. (2020). Monolithic Metal-Containing TiO_2_ Aerogels Assembled from Crystalline Pre-Formed Nanoparticles as Efficient Photocatalysts for H_2_ Generation. Appl. Catal. B.

[35] Williams P.A., Ireland C.P., King P.J., Chater P.A., Boldrin P., Palgrave R.G., Claridge J.B., Darwent J.R., Chalker P.R., Rosseinsky M.J. (2012). Atomic Layer Deposition of Anatase TiO_2_ Coating on Silica Particles: Growth, Characterization and Evaluation as Photocatalysts for Methyl Orange Degradation and Hydrogen Production. J. Mater. Chem..

[36] Ljubas D., Smoljanić G., Juretić H. (2015). Degradation of Methyl Orange and Congo Red Dyes by Using TiO_2_ Nanoparticles Activated by the Solar and the Solar-like Radiation. J. Environ. Manag..

[37] Morales-García Á., Macià Escatllar A., Illas F., Bromley S.T. (2019). Understanding the Interplay between Size, Morphology and Energy Gap in Photoactive TiO_2_ Nanoparticles. Nanoscale.

[38] Comparelli R., Fanizza E., Curri M.L., Cozzoli P.D., Mascolo G., Passino R., Agostiano A. (2005). Photocatalytic Degradation of Azo Dyes by Organic-Capped Anatase TiO_2_ Nanocrystals Immobilized onto Substrates. Appl. Catal. B.

[39] Kokilavani S., Alaraidh I.A., Okla M.K., Chandran P., Mohebaldin A., Soufan W., AL-ghamdi A.A., Abdel-Maksoud M.A., AbdElgawad H., Thomas A.M. (2022). Efficient Photocatalytic Degradation of Methyl Orange and Malachite Green by Ag_3_PO_4_ Decorated BiOBr Nanoflower under Visible Light: Performance Evaluation, Mechanism Insights and Toxicology of the by-Products. J. Alloys Compd..

[40] Putri R.A., Safni S., Jamarun N., Septiani U., Kim M.-K., Zoh K. (2019). Kinetics Studies on Photodegradation of Methyl Orange in the Presence of C-N-Codoped TiO_2_ Catalyst. Egypt. J. Chem..

[41] Baiocchi C., Brussino M.C., Pramauro E., Prevot A.B., Palmisano L., Marcì G. (2002). Characterization of Methyl Orange and Its Photocatalytic Degradation Products by HPLC/UV–VIS Diode Array and Atmospheric Pressure Ionization Quadrupole Ion Trap Mass Spectrometry. Int. J. Mass. Spectrom..

[42] Kim K.-S., Kam S.K., Mok Y.S. (2015). Elucidation of the Degradation Pathways of Sulfonamide Antibiotics in a Dielectric Barrier Discharge Plasma System. Chem. Eng. J..

[43] Liu T., Wang L., Lu X., Fan J., Cai X., Gao B., Miao R., Wang J., Lv Y. (2017). Comparative Study of the Photocatalytic Performance for the Degradation of Different Dyes by ZnIn_2_S_4_: Adsorption, Active Species, and Pathways. RSC Adv..

[44] Mills A., Williams G. (1987). Methyl Orange as a Probe of the Semiconductor–Electrolyte Interfaces in CdS Suspensions. J. Chem. Soc. Faraday Trans..

[45] Osawa R.A., Barrocas B.T., Monteiro O.C., Conceição Oliveira M., Florêncio M.H. (2019). Photocatalytic Degradation of Amitriptyline, Trazodone and Venlafaxine Using Modified Cobalt-Titanate Nanowires under UV–Vis Radiation: Transformation Products and in Silico Toxicity. Chem. Eng. J..

[46] Ismail L., Rifai A., Ferronato C., Fine L., Jaber F., Chovelon J.-M. (2016). Towards a Better Understanding of the Reactive Species Involved in the Photocatalytic Degradation of Sulfaclozine. Appl. Catal. B.

[47] Dirany A., Sirés I., Oturan N., Özcan A., Oturan M.A. (2012). Electrochemical Treatment of the Antibiotic Sulfachloropyridazine: Kinetics, Reaction Pathways, and Toxicity Evolution. Environ. Sci. Technol..

[48] Fabiańska A., Białk-Bielińska A., Stepnowski P., Stolte S., Siedlecka E.M. (2014). Electrochemical Degradation of Sulfonamides at BDD Electrode: Kinetics, Reaction Pathway and Eco-Toxicity Evaluation. J. Hazard. Mater..

[49] Vale M., Marques A.C. (2023). Mechanistic Study of the Formation of Multicomponent Oxide Porous Microspheres (MICROSCAFS^®^) by Cryo-Scanning Electron Microscopy. Gels.

[50] Landi S., Segundo I.R., Freitas E., Vasilevskiy M., Carneiro J., Tavares C.J. (2022). Use and Misuse of the Kubelka-Munk Function to Obtain the Band Gap Energy from Diffuse Reflectance Measurements. Solid State Commun..

[51] Makuła P., Pacia M., Macyk W. (2018). How To Correctly Determine the Band Gap Energy of Modified Semiconductor Photocatalysts Based on UV–Vis Spectra. J. Phys. Chem. Lett..

[52] Barrocas B., Chiavassa L.D., Conceição Oliveira M., Monteiro O.C. (2020). Impact of Fe, Mn Co-Doping in Titanate Nanowires Photocatalytic Performance for Emergent Organic Pollutants Removal. Chemosphere.

[53] Barrocas B.T., Oliveira M.C., Nogueira H.I.S., Fateixa S., Monteiro O.C. (2019). Ruthenium-Modified Titanate Nanowires for the Photocatalytic Oxidative Removal of Organic Pollutants from Water. ACS Appl. Nano Mater..

[54] Osawa R.A., Barrocas B.T., Monteiro O.C., Oliveira M.C., Florêncio M.H. (2019). Photocatalytic Degradation of Cyclophosphamide and Ifosfamide: Effects of Wastewater Matrix, Transformation Products and in Silico Toxicity Prediction. Sci. Total Environ..

