# Supplementary material for: Robust Photocatalytic MICROSCAFS® with Interconnected Macropores for Sustainable Solar-Driven Water Purification
Source: Int J Mol Sci. 2024 May 29;25(11):5958. doi: 10.3390/ijms25115958 (PMC11172857; doi:10.3390/ijms25115958)
Supplement: Supplementary file 1 [file ijms-25-05958-s001.zip › ijms-2997510-supplementary.pdf]

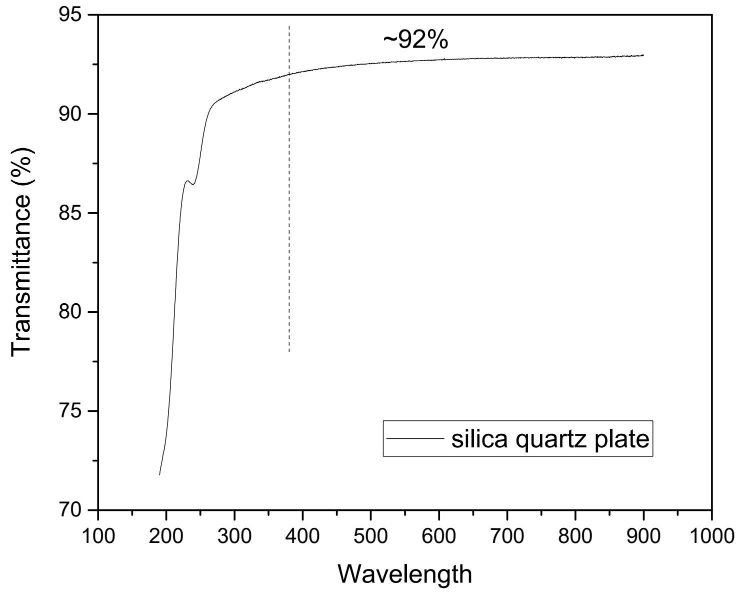

Figure S1. Transmission spectrum of the silica quartz window employed in the continuous flow reactor, and also to cover the batch reactor.

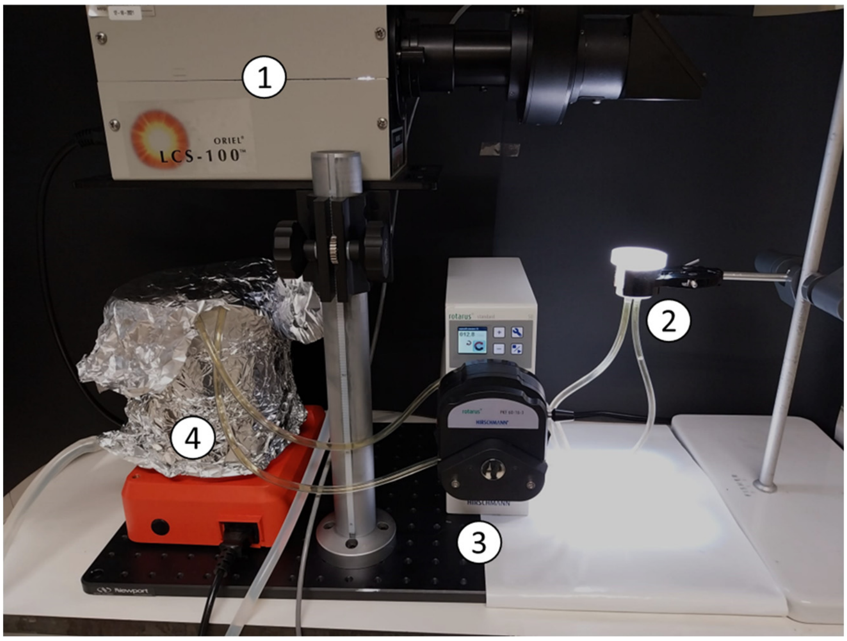

Figure S2. Continuous flow reactor with total recirculation to a tank. 1 – Solar simulator; 2 – Sample chamber (solar reactor); 3 – Peristaltic pump; 4 – MO solution stirred tank.

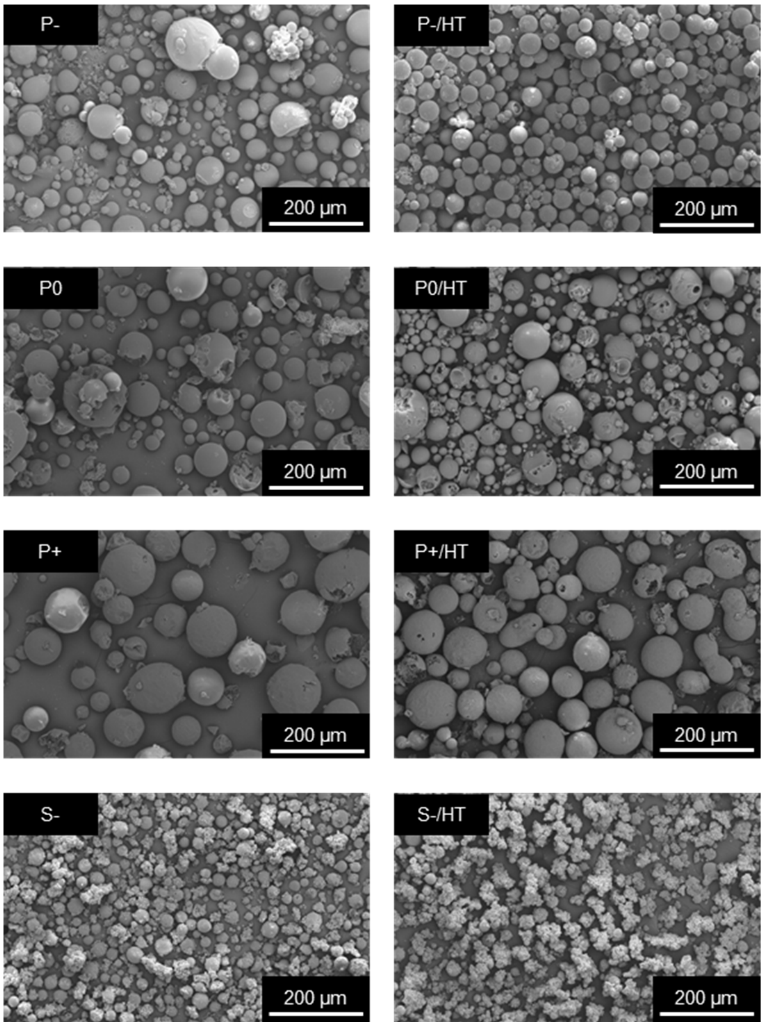

Figure S3. SEM images of the MICROSCAFS® used for the particle size distributions, before (left column) and after (right column) being heat treated at 900 °C for 30 minutes.

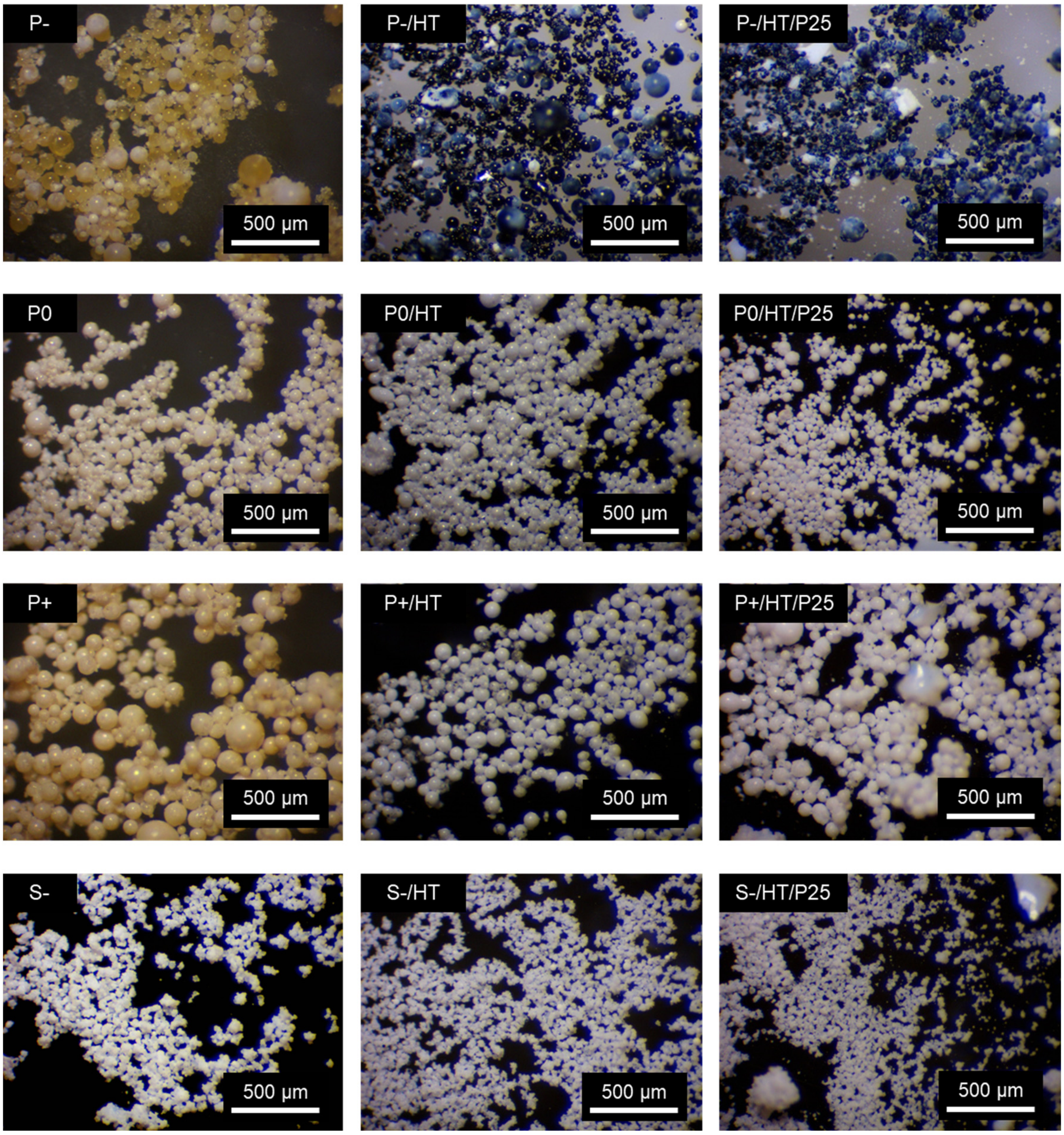

Figure S4. Optical microscopy photographs of the MICROSCAFS® in three subsequent stages: 1 (first column) – dried MICROSCAFS®; 2 - (second column) MICROSCAFS® after being heat treated at 900 °C for 30 minutes; 3 (third column) – MICROSCAFS® after being loaded with the P25 TiO_2 NPs.

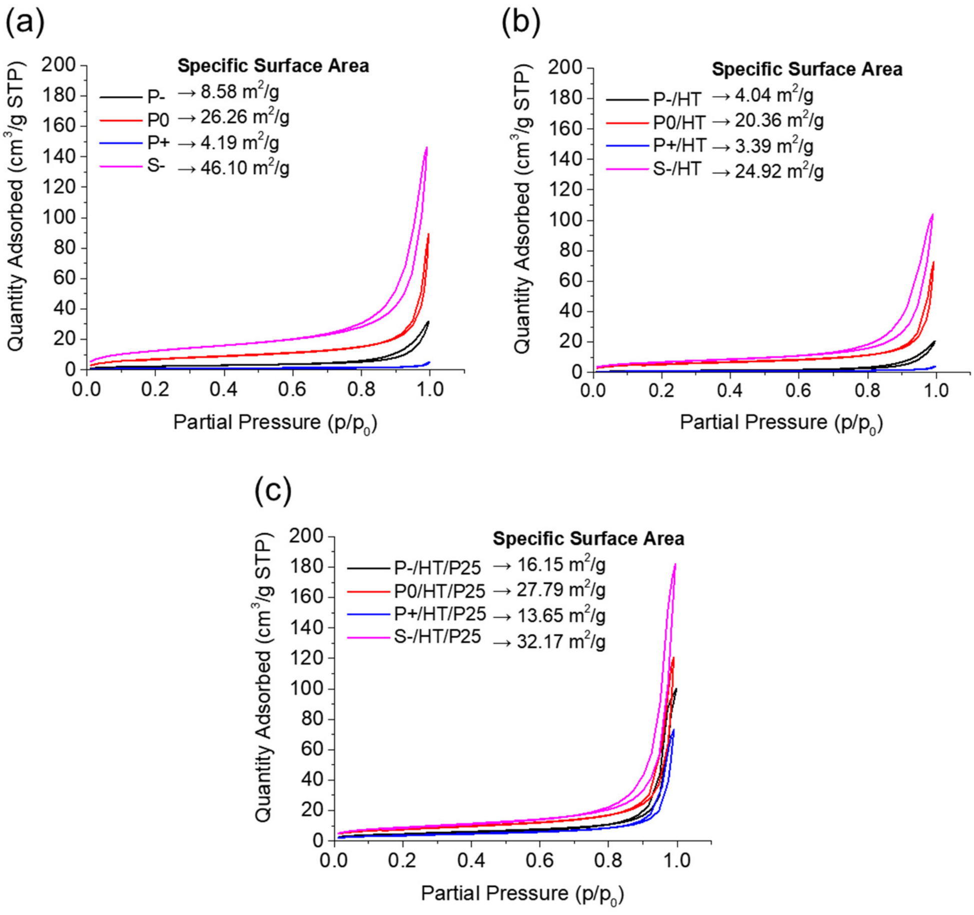

Figure S5. N_2 adsorption-desorption isotherms of the MICROSCAFS® (a) dried at 45 °C, (b) heat-treated at 900 °C and (c) loaded with P25 TiO2 NPs.

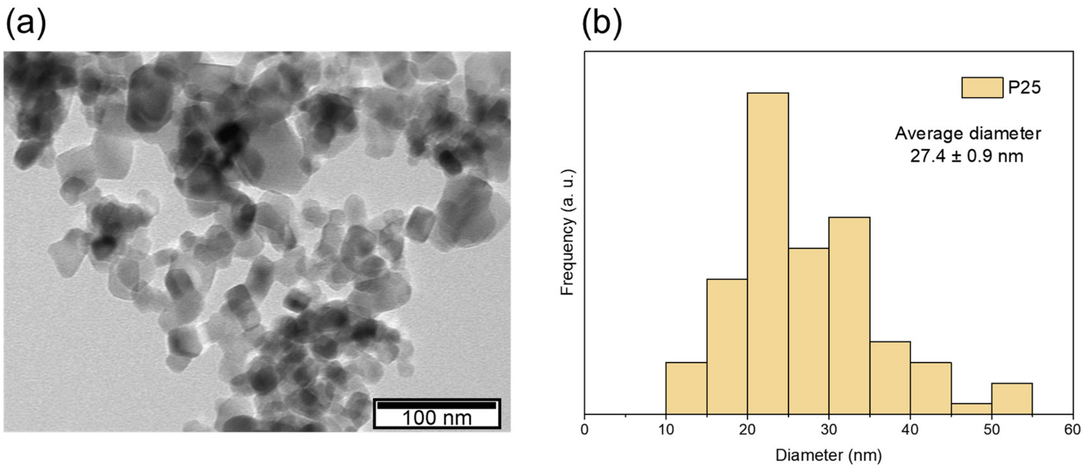

Figure S6. (a) TEM image of the P25 TiO_2 NPs; (b) their respective particle size distribution.

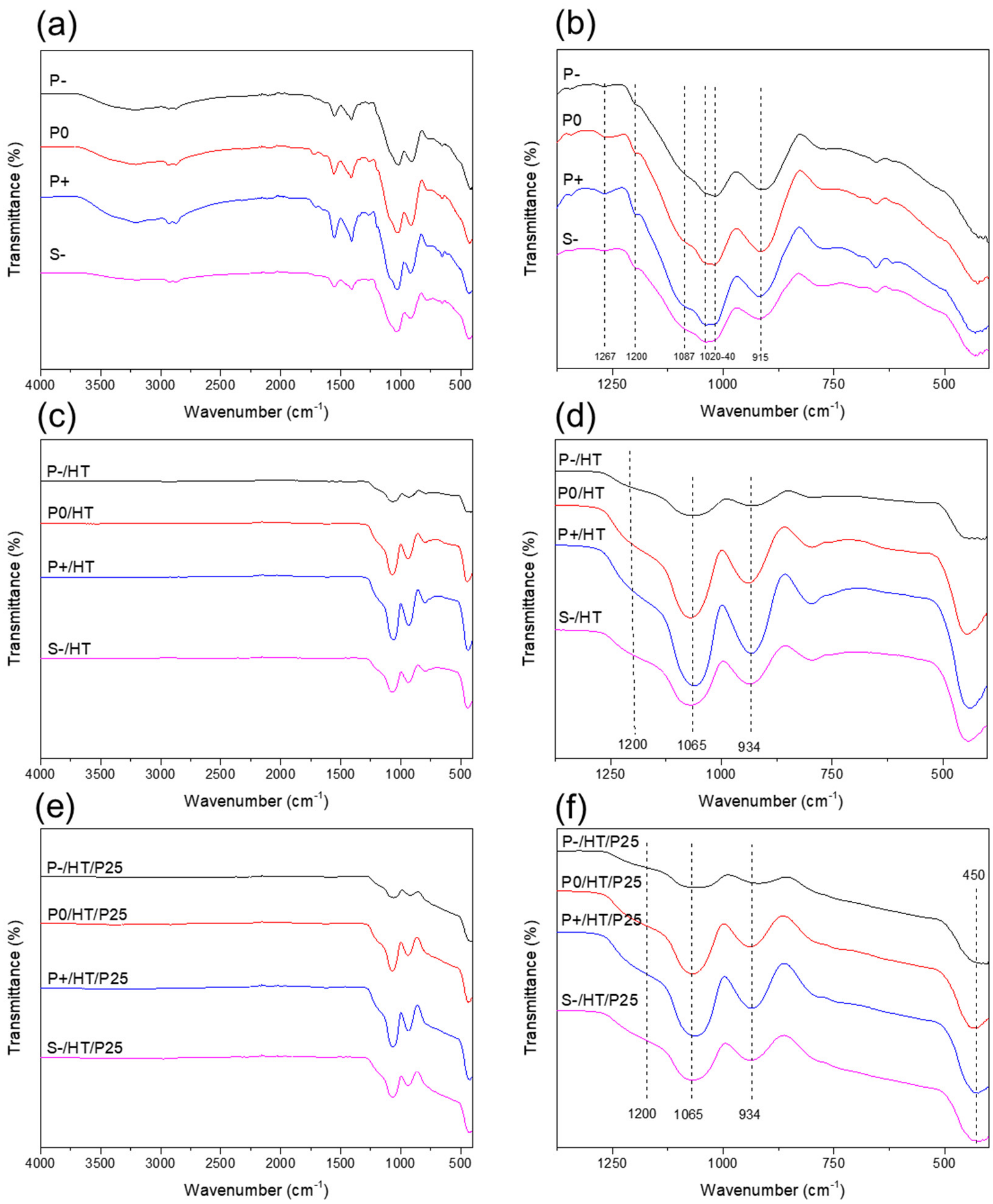

Figure S7. ATR-FTIR spectra of all the MICROSCAFS® (a, c and e) and respective magnification in the range of 1500 to 400 cm^{-1} (b, d and f).

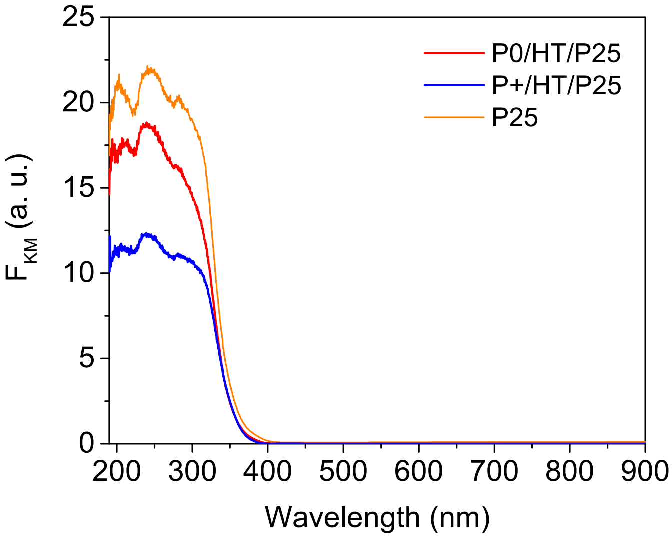

Figure S8. Kubelka-Munk transformed UV-Vis DRS absorption spectra of the P25 TiO_2 NPs loaded MICROSCAFS® and P25 TiO_2 NPs.

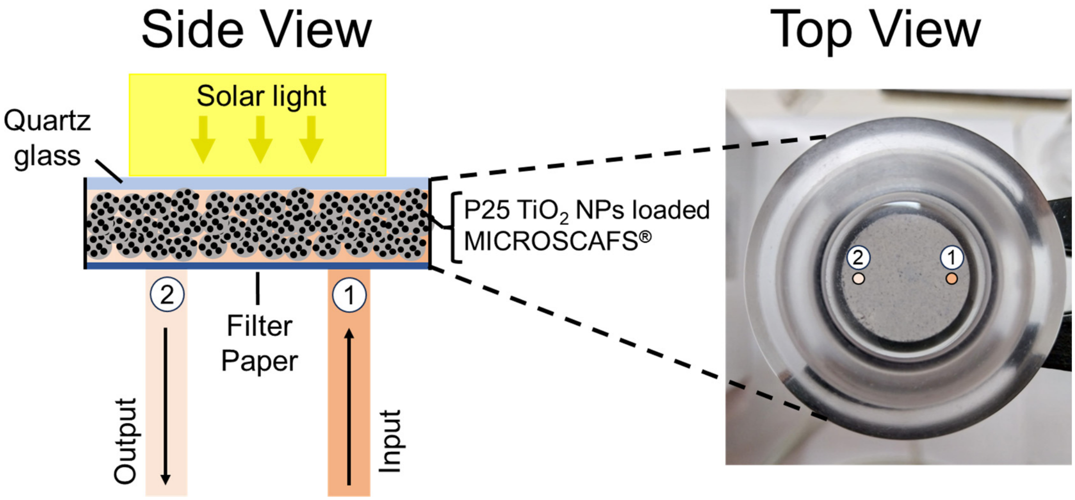

Figure S9. Side and top views of the continuous flow reactor's sample chamber.

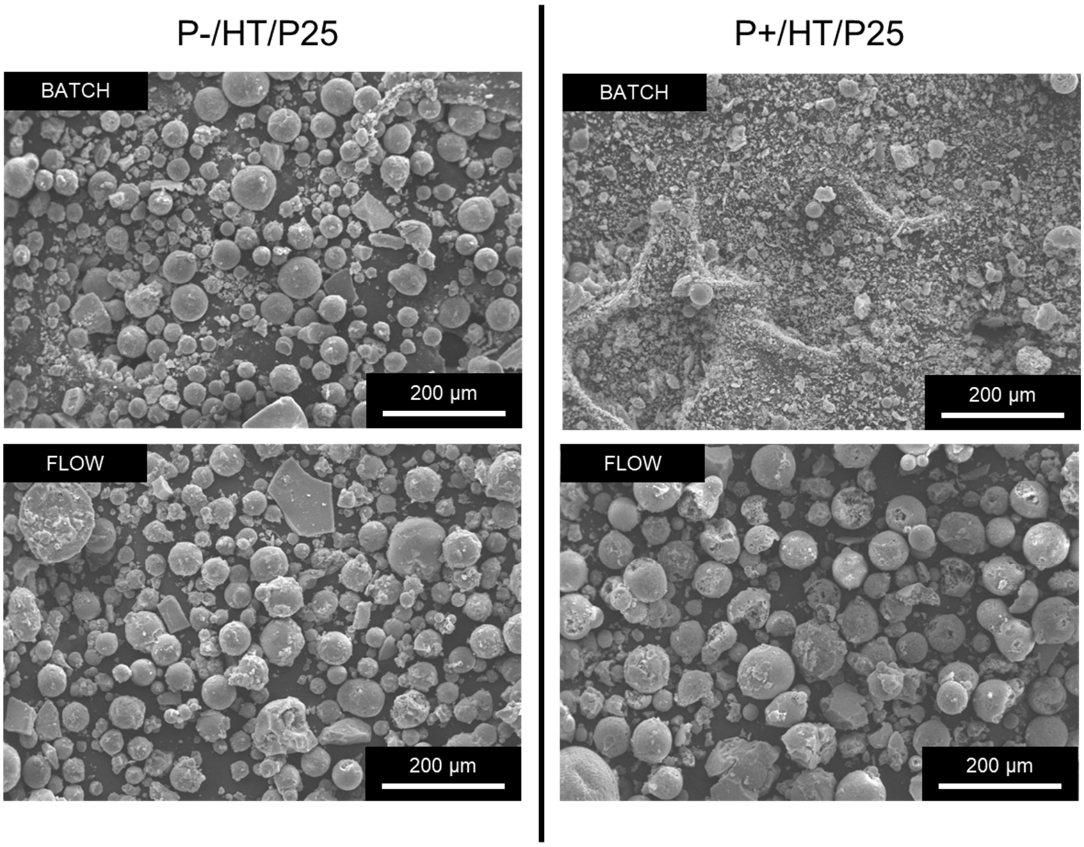

Figure S10. SEM images of the P-/HT/P25 (left column) and P+/HT/P25 (right column) photocatalytic MICROSCAFS® after one cycle in batch and in flow.

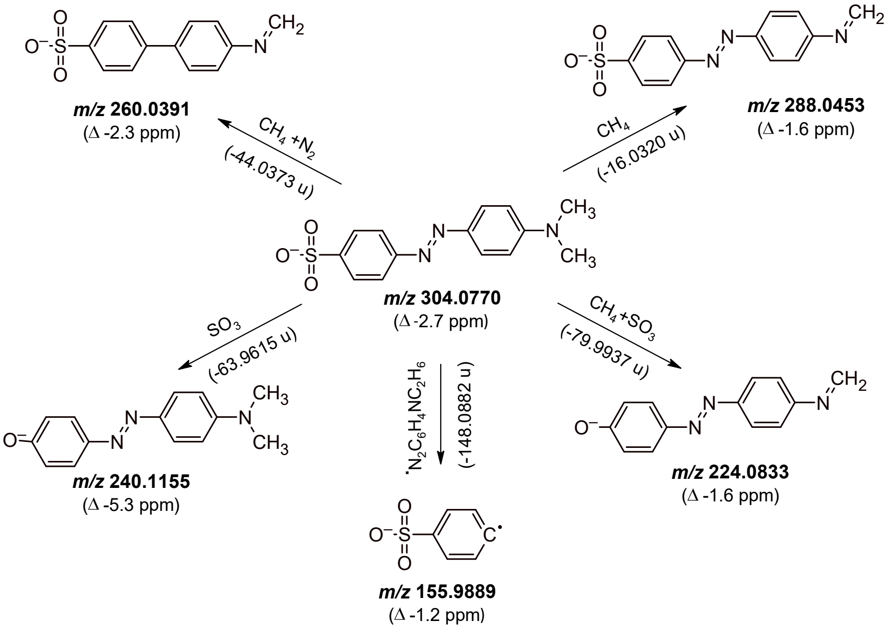

Figure S11. Proposed fragmentation mechanism for the precursor ion *m/z* 304.0770, t_R 9.5 min, assigned to the deprotonated molecule of MO.

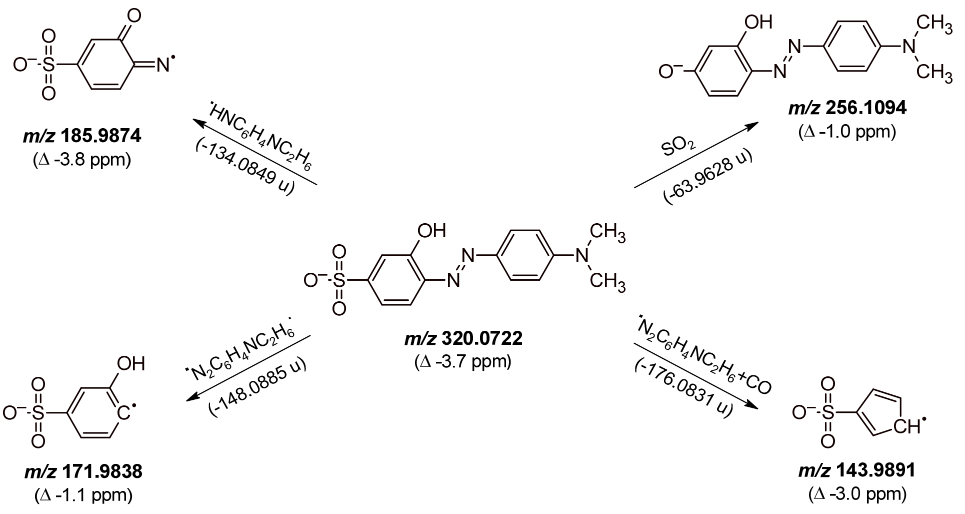

Figure S12. Proposed fragmentation mechanism for the precursor ion *m/z* 320.0712, t_R 10.9 min, attributed to the deprotonated molecule of TP320-a.

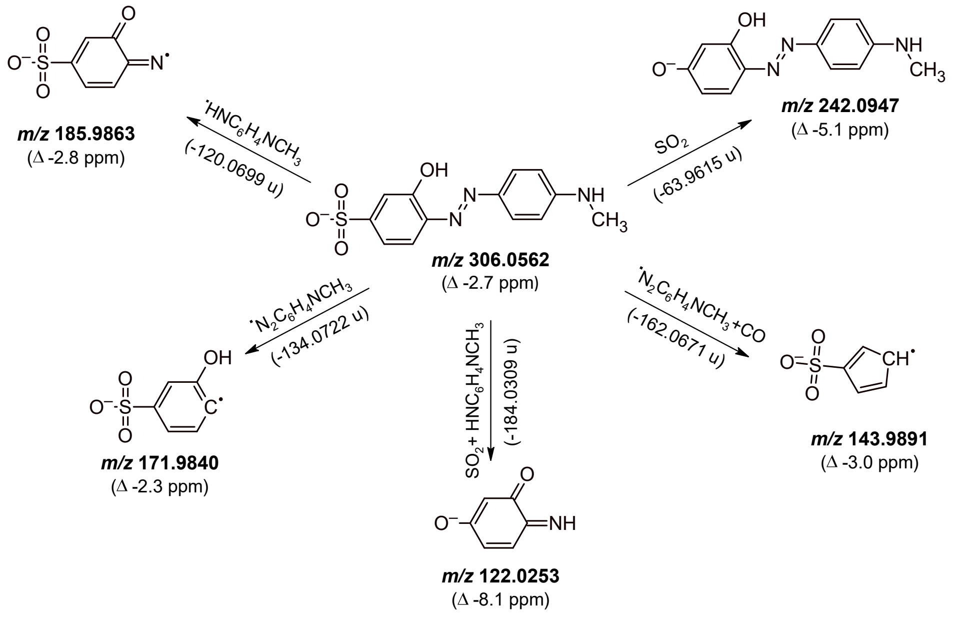

Figure S13. Proposed fragmentation mechanism for the precursor ion *m/z* 306.0562, t_R 9.0 min, attributed to the deprotonated molecule of TP306.

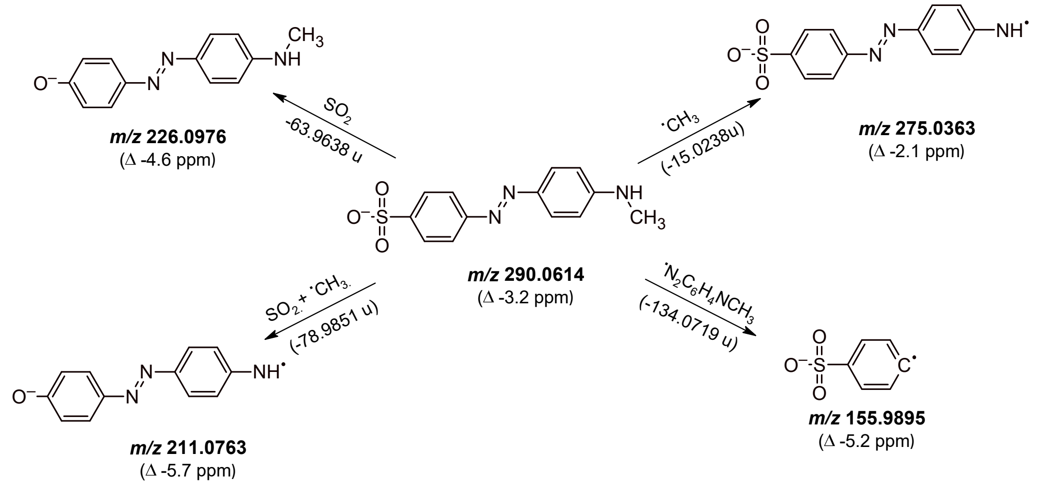

Figure S14. Proposed fragmentation mechanism for the precursor ion *m/z* 290.0614, t_R 7.8 min, attributed to the deprotonated molecule of TP290.

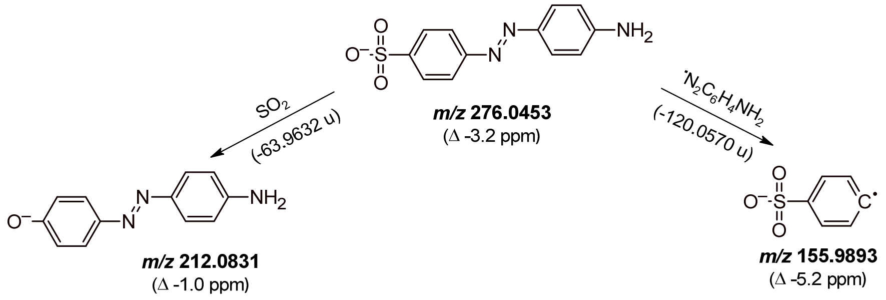

Figure S15. Proposed fragmentation mechanism for the precursor ion *m/z* 276.0453, t_R 5.8 min, attributed to the deprotonated molecule of TP276.

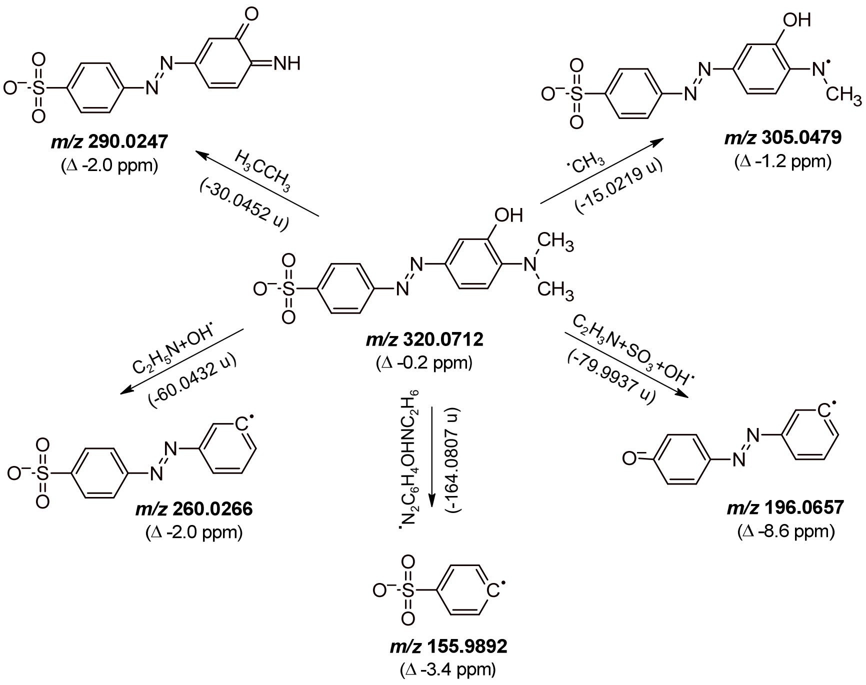

Figure S16. Proposed fragmentation mechanism for the precursor ion *m/z* 320.0712, t_R 3.9 min, assigned to the deprotonated molecule of TP320-b.

Table S1. Particle size distribution data of the treated and non-heat-treated MICROSCAFS®.

Sample acronym	D (0.1), µm	D (0.5), µm	D (0.9), µm	Span
P-	20.16	29.61	50.56	1.03
P0	22.58	33.99	65.36	1.26
P+	43.68	72.29	107.52	0.88
S-	19.02	24.50	34.13	0.62
P-/HT	18.62	34.29	72.86	1.58
P0/HT	16.24	26.23	50.99	1.32
P+/HT	31.88	56.12	83.87	0.93
S-/HT	14.29	17.90	25.07	0.60

Table S2. EDS atomic concentration data of the MICROSCAFS®. Ti/Si is the atomic % ratio.

Sample acronym	EDS atomic concentration (%)			Ti/Si
	O	Si	Ti	
P-	67.37 ± 0.59	25.68 ± 0.73	6.94 ± 0.31	0.272 ± 0.017
P0	67.53 ± 0.44	25.0 4± 0.60	7.43 ± 0.31	0.298 ± 0.018
P+	63.95 ± 0.35	27.94 ± 0.27	8.11 ± 0.10	0.290 ± 0.002
S-	62.00 ± 0.35	34.4 ± 0.2	3.6 ± 0.2	0.106 ± 0.005
P-/HT	61.22 ± 0.23	30.50 ± 0.08	8.28 ± 0.26	0.272 ± 0.009
P0/HT	60.32 ± 0.03	30.38 ± 0.07	9.29 ± 0.05	0.306 ± 0.002
P+/HT	60.25 ± 0.19	30.31 ± 0.21	9.43 ± 0.14	0.311 ± 0.006
S-/HT	60.24 ± 0.20	32.97 ± 0.07	6.78 ± 0.005	0.206 ± 0.005
P-/HT/P25	70.26 ± 0.84	12.51 ± 0.57	17.17 ± 0.89	1.40 ± 0.11
P0/HT/P25	69.05 ± 0.30	16.52 ± 0.29	14.44 ± 0.30	0.88 ± 0.03
P+/HT/P25	71.17 ± 0.26	18.00 ± 0.75	10.84 ± 0.73	0.62 ± 0.07
S-/HT/P25	74.31 ± 0.57	17.79 ± 0.61	7.89 ± 0.27	0.45 ± 0.03

Table S3. FTIR peak intensity ratio $\frac{I_{Ti-O-Ti}}{I_{Si-O-Si}}$

**Sample**	$\frac{\boldsymbol{I_{450\ cm^{-1}}}}{\boldsymbol{I_{1065\ cm^{-1}}}}$
P-/HT	1.04
P0/HT	0.80
P+/HT	1.28
S-/HT	1.20
P-/HT/P25	3.60
P0/HT/P25	1.60
P+/HT/P25	1.48
S-/HT/P25	1.87

Table S4. Experimental conditions, MO degradation and kinetic rate constants (k or k_{app}) of supported TiO_2 photocatalyst – comparison of the present study with the literature.

**Supported photocatalyst system**	**Radiation source**	**Reactor type and pH**	**Reactor volume and initial concentration of MO solution**	**Photocatalyst (active phase) / pollutant mass ratio**	**MO degradation at 464 nm**	**k (batch) or k_{app} (flow) (mL min^{-1} mg^{-1})**	Ref., year
P25 TiO_2 NPs supported in SiO_2-TiO_2 MICROSCAFS® (11.5 mg batch; 46 mg flow) (23 wt% loading)	Solar simulator Xe lamp, 100 W, 1000 W/m² (1 sun)	Batch pH=7	50 mL, 10 mg/L	23	87% in 120 min.	(6.45 ± 0.08) × 10^{-2}	Present study
		Flow pH=7	0.47 mL, 10 mg/L	23	29% in 360 min.	(4.19 ± 0.7) × 10^{-3}	
Commercial anatase TiO_2 (11 mg, 2-3 μm) supported in biochar (75% wt% loading)	Hg lamp, 500 W	Batch pH = 6.3	30 mL, 60 mg/L	6	~99% in 60 min.	1.39 × 10^{-1}	[6], 2023
Synthesized anatase TiO_2 (66 mg) supported in SiO_2 MICROSCAFS® (22 wt% loading)	Solar simulator Xe lamp, 100 W, 1000 W/m² (1 sun)	Flow pH=7	100 mL, 20 mg/L	33	30% in 375 min.	1.36 × 10^{-3}	[7], 2021
Synthesized anatase TiO_2 (2 mg) supported on natural clays (~11 wt% loading)	Xe lamp, 500 W	Batch pH=n/d	20 mL, 10 mg/L	10	90% in 150 min.	1.20 × 10^{-1}	[8], 2019
Synthesized anatase TiO_2 (19 mg) supported on cellulose nanofibrils (17 wt% loading)	Solar simulator Xe lamp, 100 W, 1000 W/m² (1 sun)	Flow pH=7	100 mL, 20 mg/L	10	21% in 200 min.	5.79 × 10^{-3}	[9], 2018
P25 TiO_2 (16 mg) supported in porous PMMA wafers (loading n/d)	UV-LED	Batch pH =n/d	60 mL, 10 mg/L	27	28% in ~30 min.	4.08 × 10^{-2}	[10], 2015
P25 TiO_2 (50 mg) supported on fly ash (25 wt% loading)	Black light lamps, 320-440 nm	Batch pH=8.5	200 mL, 3.27 mg/L	76	50% in 170 min.	6.40 × 10^{-3}	[12], 2014
Anatase TiO_2 layer (~9.8 mg) deposited on 500 nm SiO_2 spheres (10 wt% loading)	Xe lamp, 300 W	Batch pH=n/d	90 mL, 20 mg/L	5	90% in 120 min.	1.65 × 10^{-1}	[35], 2012
P25 TiO_2 (10 mg) supported on a PVA film (loading n/d)	UV 300 nm lamps, 6x8 W, 90 ±10 μW/cm²	Batch pH=n/d	10 mL, 15 mg/L	67	~100% in 300 min.	1.30 × 10^{-2}	[11], 2012

Synthesis of the MICROSCAFS®

The synthesis of silica-titania (ST) MICROSCAFS® consisted of two main steps: step 1 – silane precursors hydrolysis and simultaneous complexation of the titania precursor, and step 2 – alkaline condensation in the water droplets of the emulsion medium and MICROSCAFS® formation.

In step 1, 16.3 mL of TEOS, 13.8 mL of GPTMS, and 11.6 mL of HCl 0.28 M (aq. solution) were mixed in a closed container under constant vigorous stirring (hydrolysate solution), for 65 minutes. In parallel, 10 mL of TiPOT and 8 mL of glacial acetic acid were mixed in a different container and left under stirring at room temperature (RT) for 45 minutes.

In step 2, the mixture of both hydrolysate solution and chelated TiPOT solution (silica-titania precursor solution) was transferred to a three-neck round bottom flask, which contained a W/O emulsion, at 50 °C. The emulsion consists of 45 g of water, 114 mL of decahydronaphthalene, and 6 mL of Span® 80 previously mixed using a high energy dispersing instrument IKA T18 digital ULTRA-TURRAX® (IKA, Staufen, Germany) at 13 000 rpm for 10 minutes. This was followed by the addition of 16 mL of ammonia 25% aqueous solution at 600 rpm of mechanical stirring. Finally, after 1 hour and 30 minutes, the formed MICROSCAFS® were collected through vacuum assisted filtration with acetone and then dried at 45 °C overnight. The MICROSCAFS® synthesis was relatively straightforward and quick (2 hours and 35 minutes) and it required no more than 50 °C to reach solid porous microspheres.

Preparation of the photocatalytic MICROSCAFS®

First, all the MICROSCAFS® were heat-treated for 30 min at 900 °C, to confer an inorganic nature to the support material and provide higher mechanical, chemical and thermal stability during the final application. Sample S-/HT was sieved and the fraction between 25-45 μm of diameter was collected and used in the subsequent studies. All the other samples were sieved <354 μm to ensure the absence of any agglomerate of MICROSCAFS® in the tests. Then, 372 mg of MICROSCAFS® were placed in 2.1 mL of a 62.5 mg mL^{-1} aqueous dispersion of P25 TiO_2 NPs in a small glass vial, targeting at a loading of 26wt% relative to the total weight of MICROSCAFS® and P25 TiO_2 NPs. This dispersion was submitted to ultrasound sonication for 2 minutes, dried at 60 °C for 15 h, and finally subjected to a heat treatment at 500 °C for 1 hour. P25 TiO_2 NPs are driven into the pores of the MICROSCAFS® by capillary action, and chemical bonds (e.g. Si-O-Ti) are formed during the subsequent heat treatment.
